# Supplementary material for: Genotypic diversity and unrecognized antifungal resistance among populations of Candida glabrata from positive blood cultures
Source: Nat Commun. 2023 Sep 22;14:5918. doi: 10.1038/s41467-023-41509-x (PMC10516878; doi:10.1038/s41467-023-41509-x)
Supplement: Supplementary file 1 — Supplementary Information [file 41467_2023_41509_MOESM1_ESM.pdf]

Genotypic diversity and unrecognized antifungal resistance among populations of *Candida glabrata* from positive blood cultures

Hassan Badrane[1], Shaoji Cheng[1], Christopher L Dupont[2], Binghua Hao[1], Eileen Driscoll[1], Kristin Morder [1], Guojun Liu [1], Anthony Newbrough[1], Giuseppe Fleres[1], Drishti Kaul[2], Josh L Espinoza[2], Cornelius J Clancy[1,3§], M. Hong Nguyen[1*§]

[1]University of Pittsburgh, Pittsburgh, Pennsylvania, USA; [2] J. Craig Venter Institute, La Jolla, CA 92037, and [3]VA Pittsburgh Healthcare System, Pittsburgh, PA USA

[§]Cornelius J. Clancy and M. Hong Nguyen contributed equally to this paper

* Corresponding author

Description of Supplementary file

SUPPLEMENTARY TABLES

Supplementary Table S1. Summary of genotypes and phenotypes of *C. glabrata* blood culture strains from patients L and J.

Supplementary Table S2. Biological processes enriched among non-synonymous variant-containing genes that discriminated between *C. glabrata* strains in each patient.

Supplementary Table S3. PDR1 mutations and fluconazole, voriconazole and posaconazole MICs

Supplementary Table S4. Biological processes enriched among variant-containing genes that discriminated within-patient strains (i.e., mutations found in at least one strain from a patient but not found in all strains, compared to *C. glabrata* CBS138) and within strains recovered from blood culture spiked with J1.

Supplementary Table S5. Primers used in this study

SUPPLEMENTARY FIGURES

Supplementary Fig S1. Sites of non-synonymous SNPs and indels within C. glabrata chromosomes.

Supplementary Fig S2. Whole genome heat map showing pairwise SNP distances of C. glabrata strains recovered from blood culture bottles.

Supplementary Fig S3. Chromosomal rearrangements of within-patient C. glabrata strains.

Supplementary Table S1. Summary of genotypes and phenotypes of *C. glabrata* blood culture strains from patients L and J

L strains	L1	L2	L3	L4	L5	L6	L7	L8	L9	L10
**Genotypic characterization**										
**gDNA identity** [a]	100%	99.98%	99.97%	99.98%	99.97%	99.97%	99.97%	99.97%	99.97%	99.97%
**mtDNA identity** [a]	100%	99.995%	99.995%	100%	99.995%	99.995%	100%	99.995%	99.995%	100%
***PDR1* genotype**	Wild-type	Wild-type	Wild-type	G346C	Wild-type	Wild-type	Wild-type	Wild-type	Wild-type	Wild-type
***PDR1* expression** [b]	1	0.845± 0.065	1.221± 0.094	2.263± 0.085	0.683± 0.068	0.845± 0.065	0.707± 0.103	0.689± 0.037	1.177± 0.073	0.742± 0.159
**C*DR1* expression** [b]	1	1.874± 0.278	1.745± 0.045	21.2± 0.558	0.460± 0.054	0.713± 0.032	0.681± 0.062	11.828± 0.589	1.199± 0.078	0.506± 0.157
**Phenotypic characterization**										
**Morphology on YPD**	NCV	NCV	NCV	NCV	NCV	NCV	NCV	NCV	NCV	NCV
**Hep-2 adherence** [c]	N/D	91.6±1.7 %	N/D	66.3±0. 8%	N/D	78.4±1.0%	N/D	N/D	N/D	N/D
**Antifungal susceptibility testing, MIC (μg/mL)**										
**Fluconazole**	16	32	32	>256	32	64	16	256	128	64
**Voriconazole**	0.25	0.25	0.5	8	0.5	0.5	0.25	4	0.5	0.5
**Posaconazole**	0.125	0.125	0.125	4	0.125	0.125	0.125	4	0.125	0.125
**Isavuconazole**	0.125	0.125	0.125	4	0.125	0.125	0.125	4	0.125	0.25
**Caspofungin**	0.25	0.25	0.25	0.25	0.25	0.25	0.25	0.25	0.25	0.25
**Micafungin**	0.015	0.015	0.015	0.015	0.015	0.015	0.015	0.015	0.015	0.015

[a] DNA identity compared to L1 strain
[b] Data are presented as mean ± standard error of mean relative to L1 strain
[c] Data are presented as mean ± standard error of mean

J strains	J1	J2	J3	J4	J5	J6	J7	J8	J9	J10
**Genotypic characterization**										
**gDNA identity** [a]	100%	99.96%	99.94%	99.95%	99.94%	99.94%	99.95%	99.94%	99.94%	99.94%
**mtDNA identity** [a]	100%	99.71%	99.98%	99.89%	99.95%	99.19%	99.12%	98.45%	96.89%	97.89%
EPA1	Absent	Absent	Absent	Absent	Absent	Present	Absent	Absent	Absent	Present
EPA5	Absent	Absent	Absent	Absent	Present	Absent	Present	Absent	Present	Absent
**CAGL0E00187g**	Present	Absent	Absent	Absent	Absent	Absent	Absent	Present	Absent	Present
rRNA-18S	Present	Present	Present	Present	Present	Present	Present	Present	Absent	Present
**Mt genome CN** [b]	30	30	30	30	8	35	18	18	1	13
**Phenotypic characterization**										
**Colony size** [c]	NCV	NCV	NCV	NCV	NCV*	SCV	SCV	SCV	SCV	SCV
**FACS Colony size (median, 95% CI of median)**	40,802 [40768-40,834]	39.977 [39,950-40,003]	40,620 [40,585-40,652]	40,166 [40,133-40,201]	15.699 [15,636-15,754]	14,914 [14,850-14,971]	15,487 [15,420-15,543]	14,985 [14,926-15,036]	14,036 [13,963-14,098]	12,615 [12,554-12,670]
**Color on eosin** [d]	Light pink	Light pink	Light pink	Light pink	Purple*	Purple	Purple	Purple	Purple	Purple
**YPD Doubling time** [e]	1.52±0.05	1.51±0.02	1.57±0.05	1.66±0.03	2.12±0.03	2.61±0.14	2.28±0.01	2.53±0.08	2.36±0.00	2.38±0.02
**YPG Doubling time** [f]	10.41±0.19	11.48±0.70	11.59±0.44	11.18±0.23	199.6±0.69*	202.8±52.41	240±72.26	204.9±74.77	243.2±94.84	84.08±10.46
**Antifungal susceptibility testing, MIC (µg/mL)**										
**Fluconazole**	8	4	8	4	32	>64	>64	>64	>64	>64
**Voriconazole**	0.5	0.5	0.5	0.5	2	>16	>16	>16	>16	>16
**Posaconazole**	0.125	0.25	0.125	0.125	2	>16	>16	>16	>16	>16
**Caspofungin**	0.125	0.125	0.125	0.25	0.125	0.125	0.25	0.125	0.25	0.25

[a] DNA identity compared to L1 strain

[b] Mt genome copy number estimation from sequencing depth by GENOME STRip.

[c] Colony size on YPD agar plates

[d] Color on eosin Y and trypan blue indicator plates

[e] Doubling time on YPD (mean±SEM)

[f] Doubling time on YP-glycerol in hours (mean±SEM)

*Strain J5 grew as NCV on YPD medium, but otherwise it was most consistent with SCV (e.g., morphology on eosin Y and trypan blue and growth in YP-glycerol broth.

Abbreviations: mt, mitochondria; SEM, standard error of mean; 95% CI: 95% confidence interval; YPD: yeast peptone dextrose; YP-glycerol: yeast peptone glycerol; NCV: normal colony variant; SCV: small colony variant; N/D, not determined.

Supplementary Table S2. Biological processes enriched among non-synonymous variant-containing genes that discriminated between *C. glabrata* strains in each patient.

We identified mutations that were found in at least one strain from individual patients' blood culture but not found in all strains (compared to *C. glabrata* CBS138). Corrected p-values were calculated by GO Term Finder (http://www.candidagenome.org/cgi-bin/GO/goTermFinder), which are probabilities of finding at least x number of genes out of the total n genes in the list annotated to a particular GO term versus the proportion of genes in the whole genome that are annotated to that GO term. The GO Term Finder uses a hypergeometric distribution with Bonferroni correction.

Patient	GO term	Cluster frequency	Background frequency	Corrected P-value	FDR	Genes
**C**	Adhesion to host	3.2% (4/154)	0.1% (5/5615)	5.73E-06	0.00%	*EPA6, EPA7, EPA1, EPA3, EPA11*
**D**	Cell-substrate adhesion	5.4% (5/141)	0.1% (7/5615)	7.23E-05	0.00%	*AWP14, EPA6, EPA7, EPA1, EPA3*
**I**	Cell adhesion	4.2% (7/167)	0.3% (19/5615)	0.00029	0.00%	*AWP14, EPA1, EPA11, EPA3, AWP2*, CAGL0I07293g, CAGL0K12078g
**L**	Cell adhesion	9.1% (4/44)	0.1% (6/5615)	4.96E-06	0.00%	*EPA6, EPA7, EPA3, EPA11*, CAGL0K12078g
	Adhesion of symbiont to host	4.5%(2/44)	0.1% (4/5615)	0.036	5.08%	*EPA6, PWP7*
**EF**	Cell-substrate adhesion	2.2% (5/223)	0.1% (7/5615)	0.00115	0.00%	*AWP14, EPA6, EPA7, EPA1*, CAGL0K12078g
**H**	Adhesion of symbiont to host	5.4% (3/56)	0.1% (5/5615)	0.002	0.00%	*EPA6, PWP7, AED1*
**J**	Mitochondrial translational elongation	5.8% (10/206)	1.1% (63/5615)	1.67E-06	0.00%	tM(CAU)8mt, tW(UCA)1mt, tT(UGU)4mt, tH(GUG)7mt, tE(UUC)10mt, tM(CAU)9mt, tP(UGG)8mt, tC(GCA)4mt, tL(UAA)4mt, tR(UCU)10mt
	Mitochondrial translation	5.8% (12/206)	1.1% (63/5612)	0.00081	0.00%	*VAR1, LSU,* tM(CAU)8mt, tW(UCA)1mt, tT(UGU)4mt, tH(GUG)7mt, tE(UUC)10mt, tM(CAU)9mt, tP(UGG)8mt, tC(GCA)4mt, tL(UAA)4mt, tR(UCU)10mt
	Mitochondrial gene expression	5.8% (12/206)	1.5% (82/5612)	0.014	0.80%	*VAR1, LSU,* tM(CAU)8mt, tW(UCA)1mt, tT(UGU)4mt, tH(GUG)7mt, tE(UUC)10mt, tM(CAU)9mt, tP(UGG)8mt, tC(GCA)4mt, tL(UAA)4mt, tR(UCU)10mt
	Intron homing	1.5% (3/206)	0.1% (3/5615)	0.02306	0.86%	*Cgai1, Cgai2, Cgai3*
	Cell substrate adhesion	2.4% (5/206)	1.5% (7/5615)	0.00055	0.00%	*AWP14, EPA6, EPA1, EPA3,* CAGL0K12078g
**K**	Cell- adhesion	7.4% (7/95)	0.3% (19/5615)	2.19E-06	0.00%	*AWP14, EPA6, EPA1, EPA3, AWP2, EPA11* CAGL0K12078g
**P**	Cell-substrate adhesion	5.1% (5/98	0.1% (7/ 5612)	5.01E-06	0.00%	*AWP14, EPA6, EPA1, EPA3,* CAGL0K12078g
**ST**	Cell adhesion	6.7% (10/129)	0.3% (19/5615)	2.14E-09	0.00%	*AWP14, EPA6, EPA7, EPA1, EPA3, EPA23, AWP2, EPA11, EPA13,* CAGL0K12078g

Supplementary Table S3. PDR1 mutations and fluconazole, voriconazole and posaconazole MICs

Strains	Fluconazole MIC (µg/mL)	Voriconazole MIC (µg/mL)	Posaconazole MIC (µg/mL)
**BG2**	16	0.25	2
**BG2 with ΔCg*PDR1***	4	<0.125	0.5
**BG2 with Δ*PDR1*_Reinsert WT Cg*PDR1***	16	0.5	2
**BG2 with Δ*PDR1*_Reinsert G346C Cg*PDR1***	64	1	2
**L4**	256	8	8
**L4 with Δ*PDR1***	4	<0.125	0.5
**L4 with Δ*PDR1*_Reinsert WT Cg*PDR1*, strain #1**	32	1	2
**L4 with Δ*PDR1*_Reinsert WT Cg*PDR1*, strain #2**	32	1	2
**L4 with Δ*PDR1*_Reinsert G346C Cg*PDR1*, strain #1**	256	4	>16
**L4 with Δ*PDR1*_Reinsert G346C Cg*PDR1*, strain #2**	256	4	>16

Supplementary Table S4. Biological processes enriched among variant (SNP or indel)-containing genes that discriminated between J strains.

We identified mutations that were found in at least one strain from patient J's blood culture but not found in all strains (compared to *C. glabrata* CBS138), or in at least one strain but not all strains recovered from a sterile blood culture spiked with strain J1. We identified 115 variant (SNP or indel)-containing genes that were found uniquely in strains J1-J10, and 20 variant-containing genes that were found uniquely in 10 strains recovered from the blood culture bottle spiked with J1. Sixty-nine variant-containing genes were identified in both J1-J10 and strains from the spiked blood culture. Corrected p-values were calculated by GO Term Finder (http://www.candidagenome.org/cgi-bin/GO/goTermFinder), which are probabilities of finding at least x number of genes out of the total n genes in the list annotated to a particular GO term versus the proportion of genes in the whole genome that are annotated to that GO term. The GO Term Finder uses a hypergeometric distribution with Bonferroni correction.

Process GO term	Cluster frequency	Background frequency	Corrected p-values	False discovery rate	Genes annotated to the term
**Genes identified uniquely in patient J blood culture (strains J1-J10) – N=115**					
**Mitochondrial translational elongation**	9.6% (11/115)	0.4%(24/5,612)	1.08E-10	0.00%	tM(CAU)8mt; tW(UCA)1mt; tT(UGU)4mt; tH(GUG)7mt; tE(UUC)10mt; tM(CAU)9mt; tP(UGG)8mt; tC(GCA)4mt; tL(UAA)4mt; tR(UCU)10mt; tG(UCC)3mt
**Mitochondrial translation**	11.3% (13/115)	1.1%(63/5,612)	8.19E-08	0.00%	VAR1; SSU; tM(CAU)8mt; tW(UCA)1mt; tT(UGU)4mt; tH(GUG)7mt; tE(UUC)10mt; tM(CAU)9mt; tP(UGG)8mt; tC(GCA)4mt; tL(UAA)4mt; tR(UCU)10mt; tG(UCC)3mt
**Intron homing**	2.6% (3/115)	0.1%(3/5,612)	0.003	0.00%	Cgai1; Cgai2; Cgai3
**Genes identified in both patient J blood culture (J1-J10) and spiked blood culture – N=69**					
**Adhesion of symbiont to host**	4.3% (3/69)	0.1%(7/5,612)	0.004	4.00%	*EPA1, EPA6, PWP7*
**Cell adhesion**	5.8% (4/69)	0.3%(18/5,612)	0.013	2.00%	*AWP2, EPA1, EPA3, EPA6*
**Genes identified uniquely in spiked blood culture – N=20**					
**Hexose mediated signaling**	10% (2/20)	0.2% (9/5,612)	0.067	15.00%	CAGL0J07282g, CAGL0L00627g

Supplementary Table S5. Primers used in this study

Name	Primers (5’-3’)	Experiment	References
**ChrG679k-For**	ATTACAGGGCCAAGCTAAAC	Gene deletion confirmation	This study
**ChrG685k-Rev**	CCAGTTCCGCCATCTTATTT	Gene deletion confirmation	This study
**ChrM550k-For**	AACACCTATGATGCAAGCACAAC	Gene duplication confirmation	This study
**ChrM550k-Rev**	GAAACTCTGCTGTATGTCCCCAT	Gene duplication confirmation	This study
**PDR1F1For_KpnI ***	ACGG*GGTACC* AAAGGGAGTGACAGCGAGAAT	Disruption of PDR1	This study
**PDR1F1Rev_ApI ***	CAAGTCAACGTA*GGGCCC*ACCTGGGAATACAACCCAAA	Disruption of PDR1	This study
**PDR1F2For_SacII ***	TGGGCCATCTT*CCGCGG*GGGAGATTTGAATGAACTC	Disruption of PDR1	This study
**PDR1F2Rev_SacI ***	CTCATAGATTACAC*GAGCTC*TGTGCTACAGACTGCATTGGA	Disruption of PDR1	This study
**PDR1ReinFor_KpnI ***	ACGG*GGTACC*AGCCTCCTATTCCGTGGAAA	Reinsertion of PDR1	This study
**PDR1ReinRev_ApaI ***	CAAGTCAACGTA*GGGCCC*TCATAGACATGGGTGCTGTGT	Reinsertion of PDR1	This study
**PDR1Up For**	TACCCCATATCGTATTGCCA	Disruption confirmation	This study
**PDR1Down Rev**	TCATAGACATGGGTGCTGTGT	Disruption confirmation	This study
**pMalRev**	TACGACTACATCAATGAAATCCAGACAGTC	Disruption confirmation	This study
**SAT1For**	GGCATTGACCTCTTCACGTATAAAACTAG	Disruption confirmation	This study
**Cgl 18S-SYBR-For**	TCGGCACCTTACGAGAAATCA	Gene expression	[58]
**Cgl 18S-SYBR-Rev**	CGACCATACTCCCCCCAGA	Gene expression	[58]
**Cgl ACT1-For**	CAAATATATAACAATGGATTCT	Excluding gDNA contamination	This study
**Cgl ACT1-Rev**	GAGTCCAAAACAATACCGG	Excluding gDNA contamination	This study
**CgACT1-SYBR-For**	TATTGACAACGGTTCCGG	Gene expression	[43]
**CgACT1-SYBR-Rev**	TAGAAAGTGTGATGCCAG	Gene expression	[43]
**CgCDR1-For**	CATACAAGAAACACCAAAGTCGGT	Gene expression	[58]
**CgCDR1-Rev**	GAGACACGCTTACGTTCACCAC	Gene expression	[58]
**CgPDR1-For**	TTTGACTCTGTTATGAGCGATTACG	Gene expression	[58]
**CgPDR1-Rev**	TTCGGATTTTTCTGTGACAATGG	Gene expression	[58]
**CgERG11-For**	CCACCCATTGCACTCTTTGT	Gene expression	[59]
**CgERG11-Rev**	AGAACGTGGTAGTCCCTTGG	Gene expression	[59]
**mito-FOR**	AATTCTGATTAATTTTTGTAGGAGCTAATG	Mitochondrion copy number	This study
**mito-REV**	GCAATAAATGATCCTACTGATGCTAC	Mitochondrion copy number	This study
**Act1-FOR**	TGGTCGGTATGGGTCAAAAG	Control copy number	This study
**Act1-REV**	CGTTGTAGAAAGTGTGATGCC	Control copy number	This study

***** Italic underlined sequences highlight the primer’s corresponding enzyme restriction site.

Supplementary Figure S1. Sites of non-synonymous SNPs and indels within *C. glabrata* chromosomes. Nucleotide differences discriminating within-patient strains were plotted on chromosomes and the mitochondrial genome using the kpPlotRainfall function of the R package karyoploteR. Each panel represents an individual chromosome or the mitochondrial genome. Chromosome ID and coordinates (x-axis) are shown by grey rectangles (bottom of figure). Black dots show positions of all SNPs and indels, and green diamonds show positions of non-synonymous variants. Black and green curves at the top of the figure correspond to density of all SNPs/indels and non-synonymous variants, respectively. Subtelomeric regions tend to accumulate more SNPs and indels than do other regions.

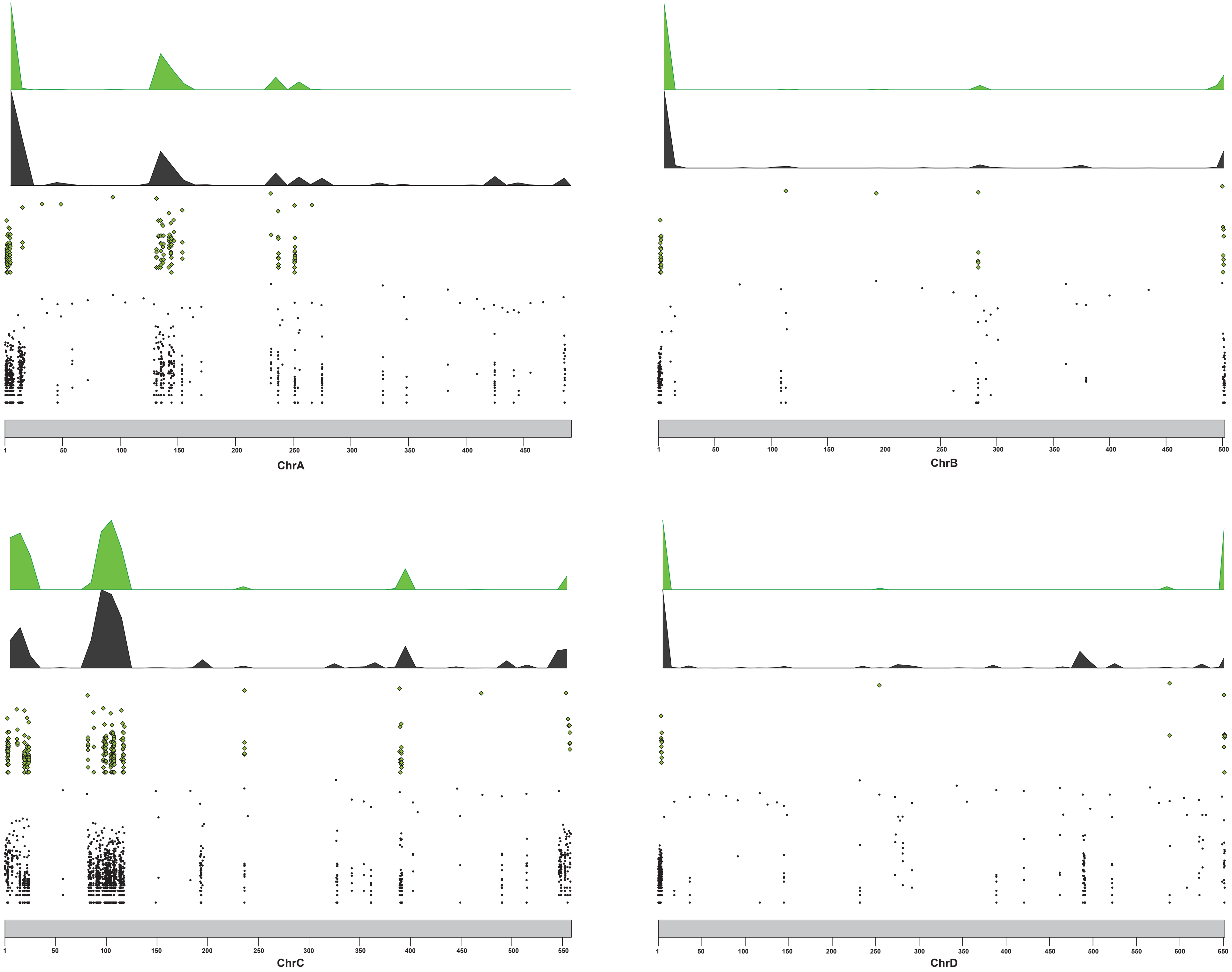
1 50 100 150 200 250 300 350 400 450
ChrA
1 50 100 150 200 250 300 350 400 450 500
ChrB
1 50 100 150 200 250 300 350 400 450 500 550
ChrC
1 50 100 150 200 250 300 350 400 450 500 550 600 650
ChrD

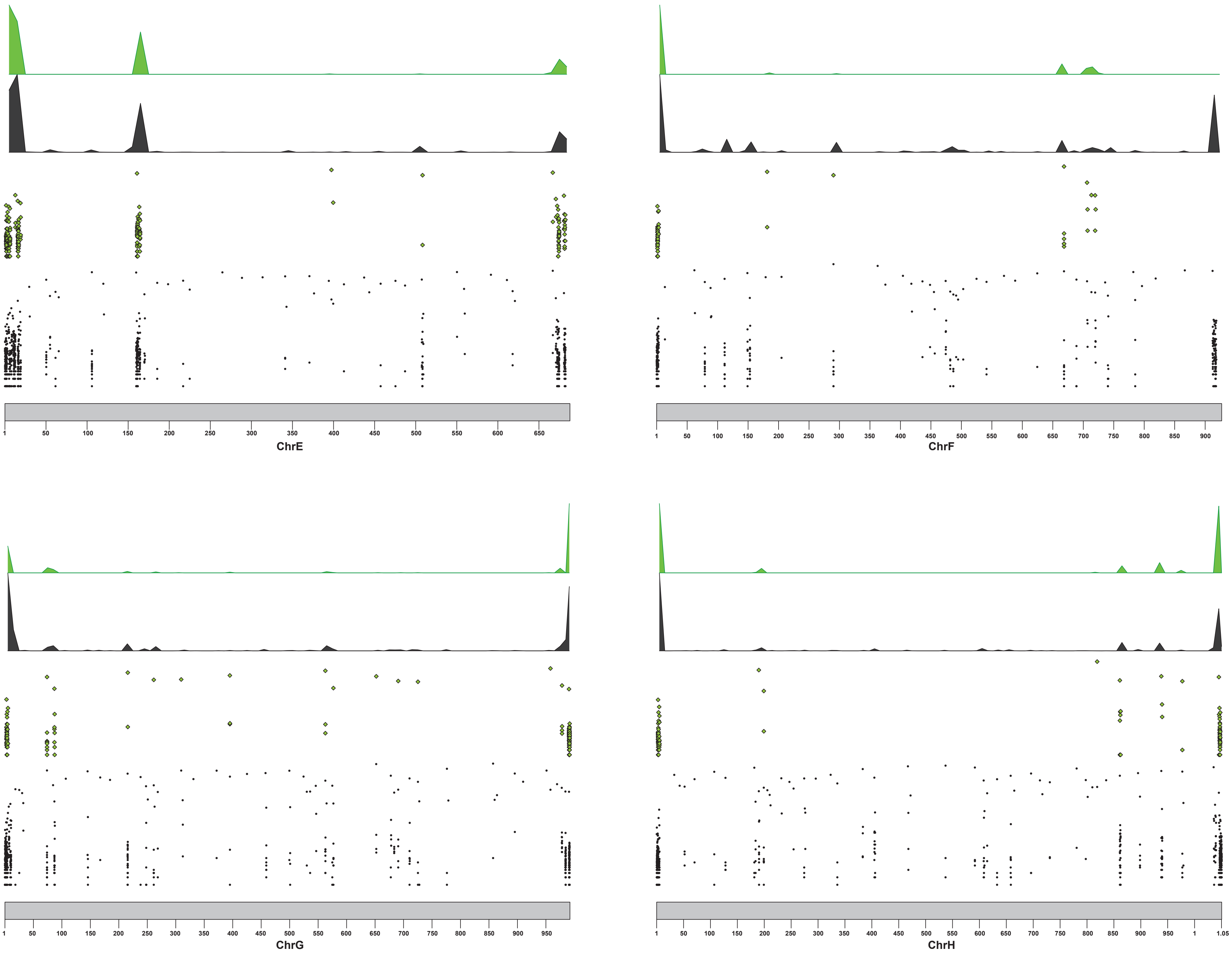
1 50 100 150 200 250 300 350 400 450 500 550 600 650
ChrE
1 50 100 150 200 250 300 350 400 450 500 550 600 650 700 750 800 850 900
ChrF
1 50 100 150 200 250 300 350 400 450 500 550 600 650 700 750 800 850 900 950
ChrG
1 50 100 150 200 250 300 350 400 450 500 550 600 650 700 750 800 850 900 950 1 1.05
ChrH

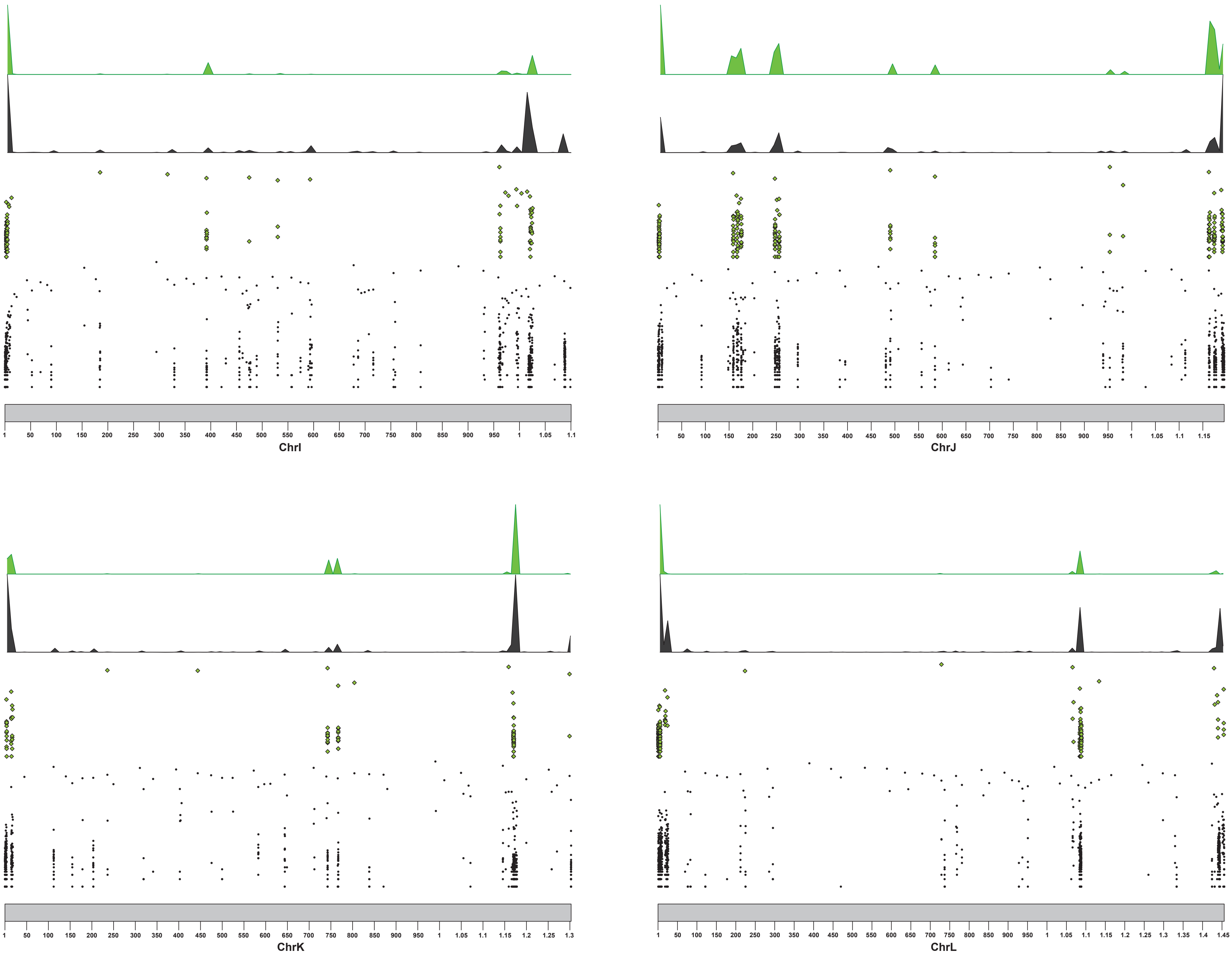
1 50 100 150 200 250 300 350 400 450 500 550 600 650 700 750 800 850 900 950 1 1.05 1.1
ChrI
1 50 100 150 200 250 300 350 400 450 500 550 600 650 700 750 800 850 900 950 1 1.05 1.1 1.15
ChrJ
1 50 100 150 200 250 300 350 400 450 500 550 600 650 700 750 800 850 900 950 1 1.05 1.1 1.15 1.2 1.25 1.3
ChrK
1 50 100 150 200 250 300 350 400 450 500 550 600 650 700 750 800 850 900 950 1 1.05 1.1 1.15 1.2 1.25 1.3 1.35 1.4 1.45
ChrL

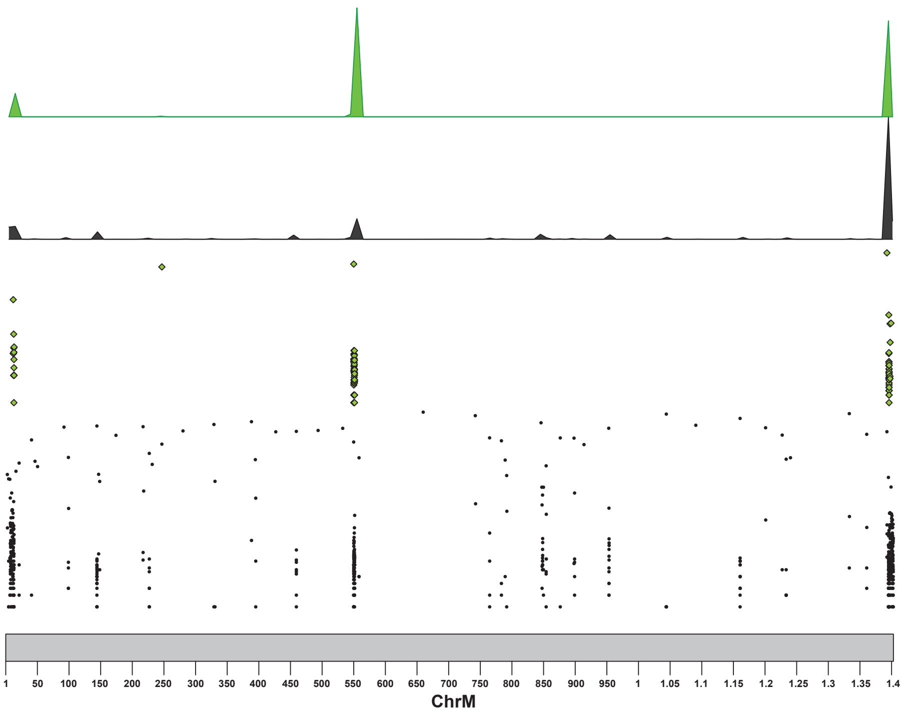
1 50 100 150 200 250 300 350 400 450 500 550 600 650 700 750 800 850 900 950 1 1.05 1.1 1.15 1.2 1.25 1.3 1.35 1.4
ChrM

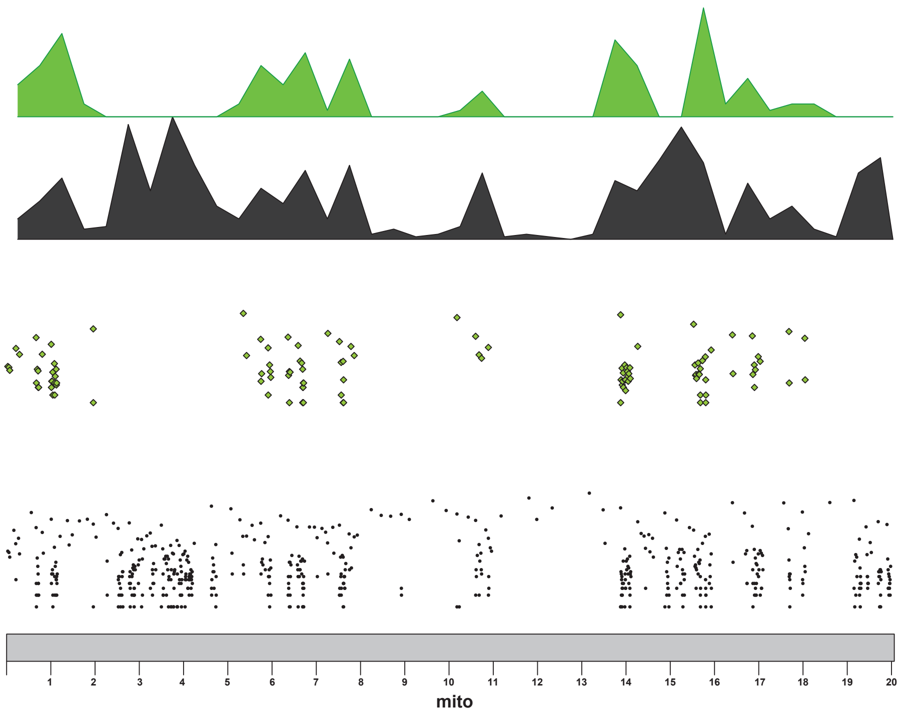
1 2 3 4 5 6 7 8 9 10 11 12 13 14 15 16 17 18 19 20
mito

Supplementary Figure S2. Whole genome heat map showing pairwise SNP distances of *C. glabrata* strains recovered from blood culture bottles. Strains from a given patient are bracketed and labeled with the appropriate letter. The SNP distance comparison of strains from individual patients are within green boxes. Strains are grouped by sequence type (ST), as indicated. Strains from patient J showed the greatest within-patient diversity.

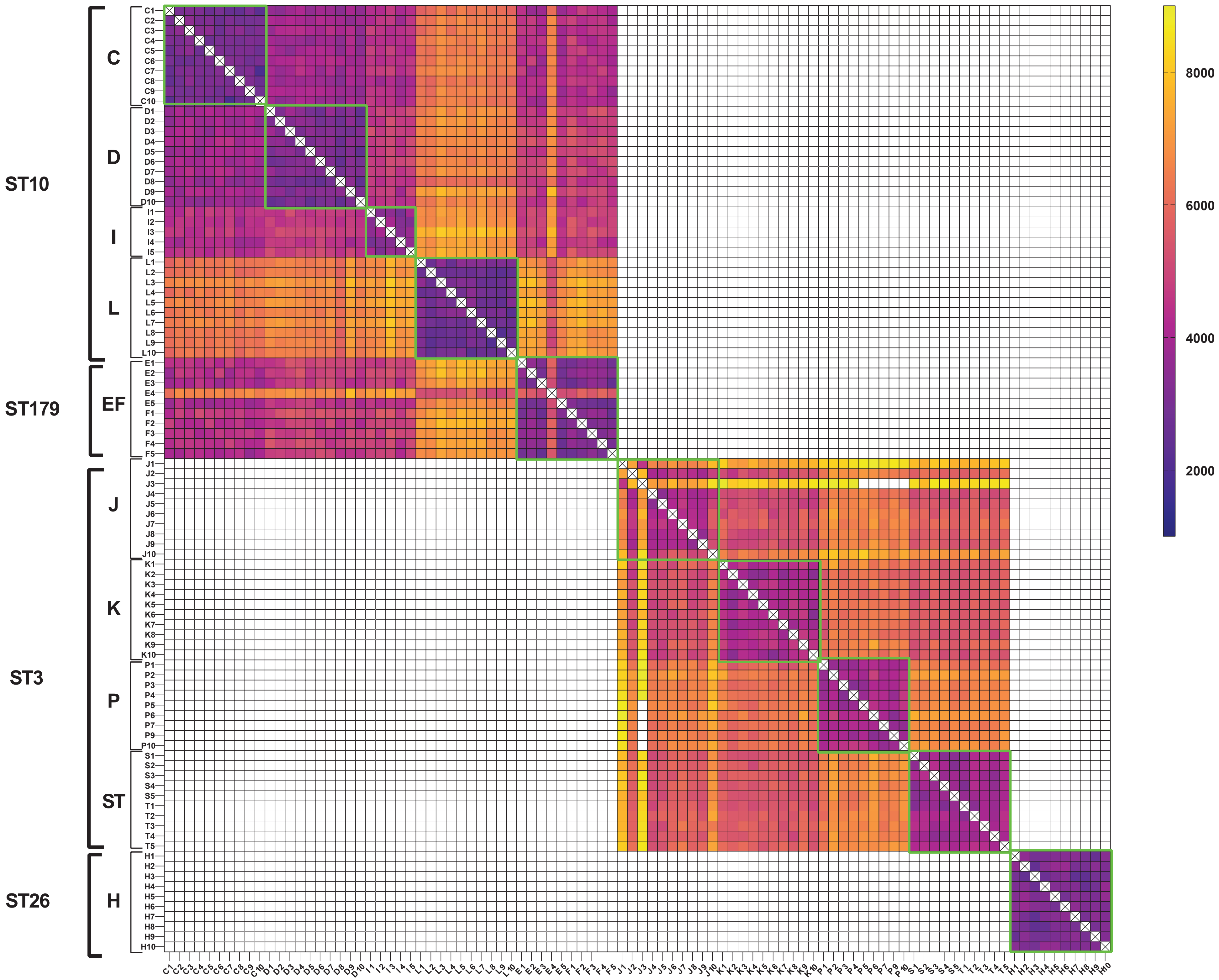
ST10
ST179
ST3
ST26
C
D
I
L
EF
J
K
P
ST
H
8000
6000
4000
2000

Supplementary Fig. S3. Chromosomal rearrangements of within-patient *C. glabrata* strains.

a Mauve genome alignments. Short reads were mapped using BWA to the index strain's long reads assembly. Pilon was used for error correction, and to polish and produce the final assembly. Assembled genomes for each patient's strains were aligned using progressiveMauve. Alignments were displayed in Mauve Alignment Viewer. Boxes delimited by short vertical lines represent contigs with colors showing matching homologous sequences that are linked by vertical or diagonal lines.

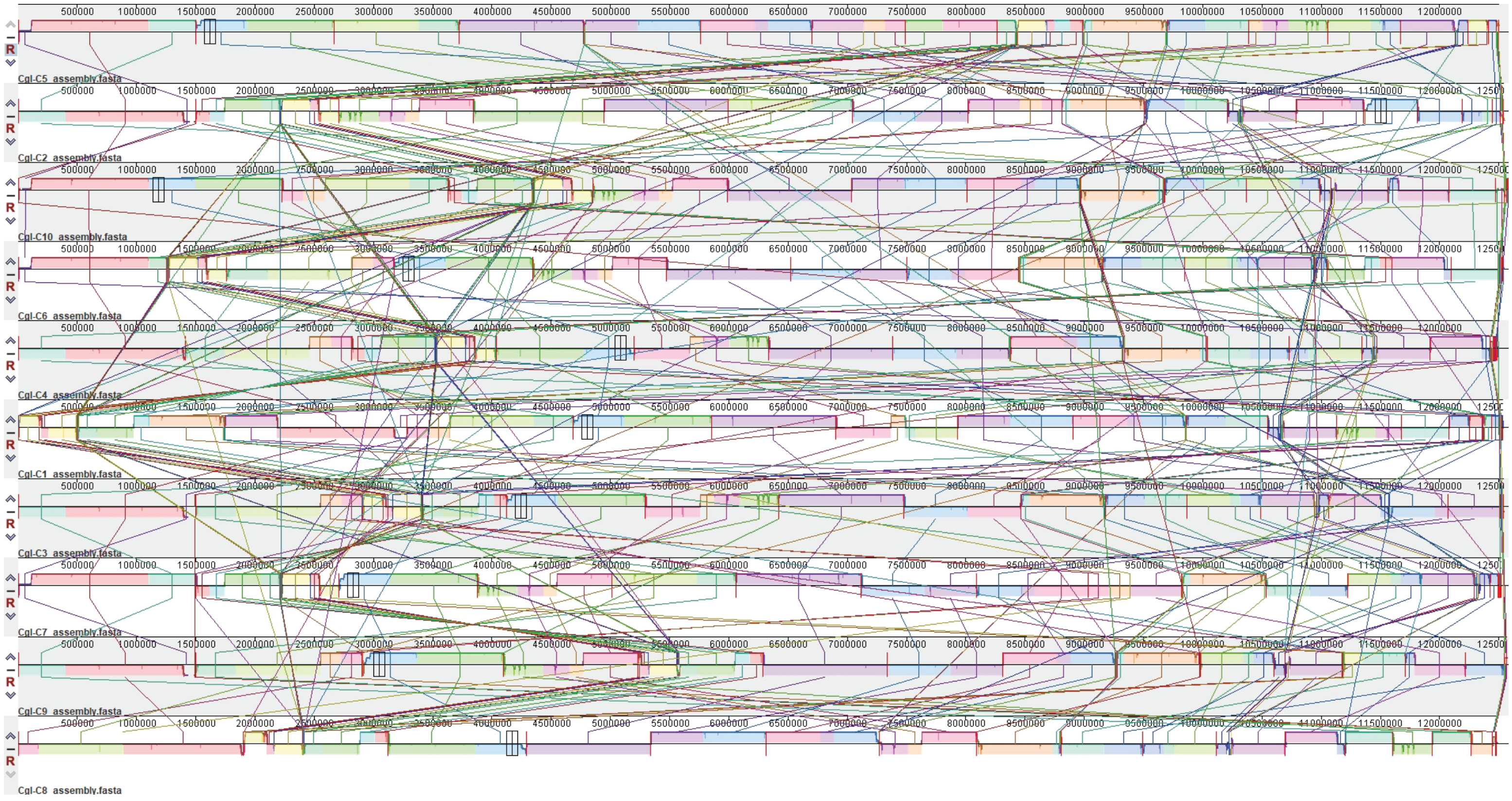
Cgl-C5_assembly.fasta
Cgl-C2_assembly.fasta
Cgl-C10_assembly.fasta
Cgl-C6_assembly.fasta
Cgl-C4_assembly.fasta
Cgl-C1_assembly.fasta
Cgl-C3_assembly.fasta
Cgl-C7_assembly.fasta
Cgl-C9_assembly.fasta
Cgl-C8_assembly.fasta

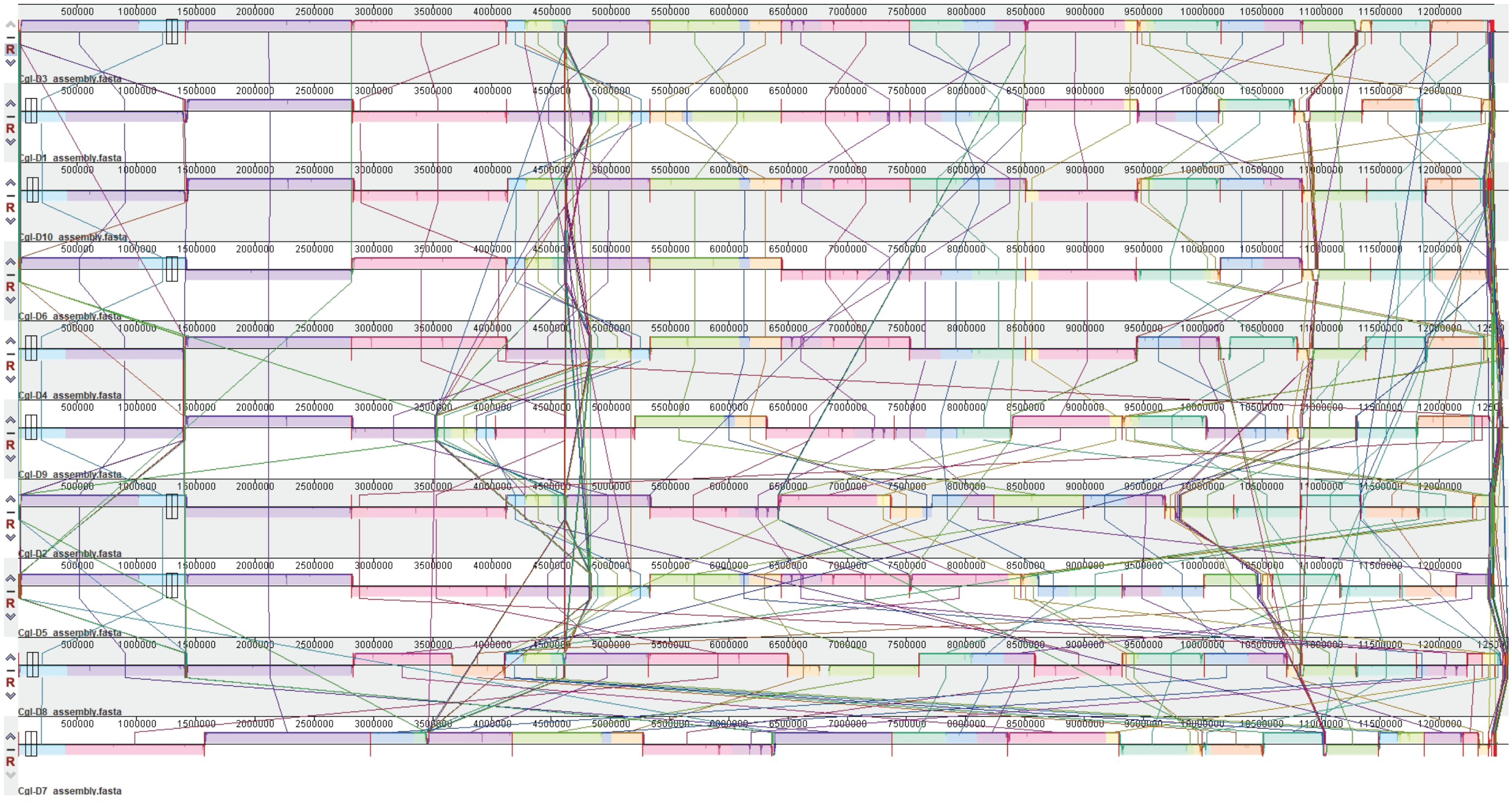
Cgl-D3_assembly.fasta
Cgl-D1_assembly.fasta
Cgl-D10_assembly.fasta
Cgl-D6_assembly.fasta
Cgl-D4_assembly.fasta
Cgl-D9_assembly.fasta
Cgl-D2_assembly.fasta
Cgl-D5_assembly.fasta
Cgl-D8_assembly.fasta
Cgl-D7_assembly.fasta

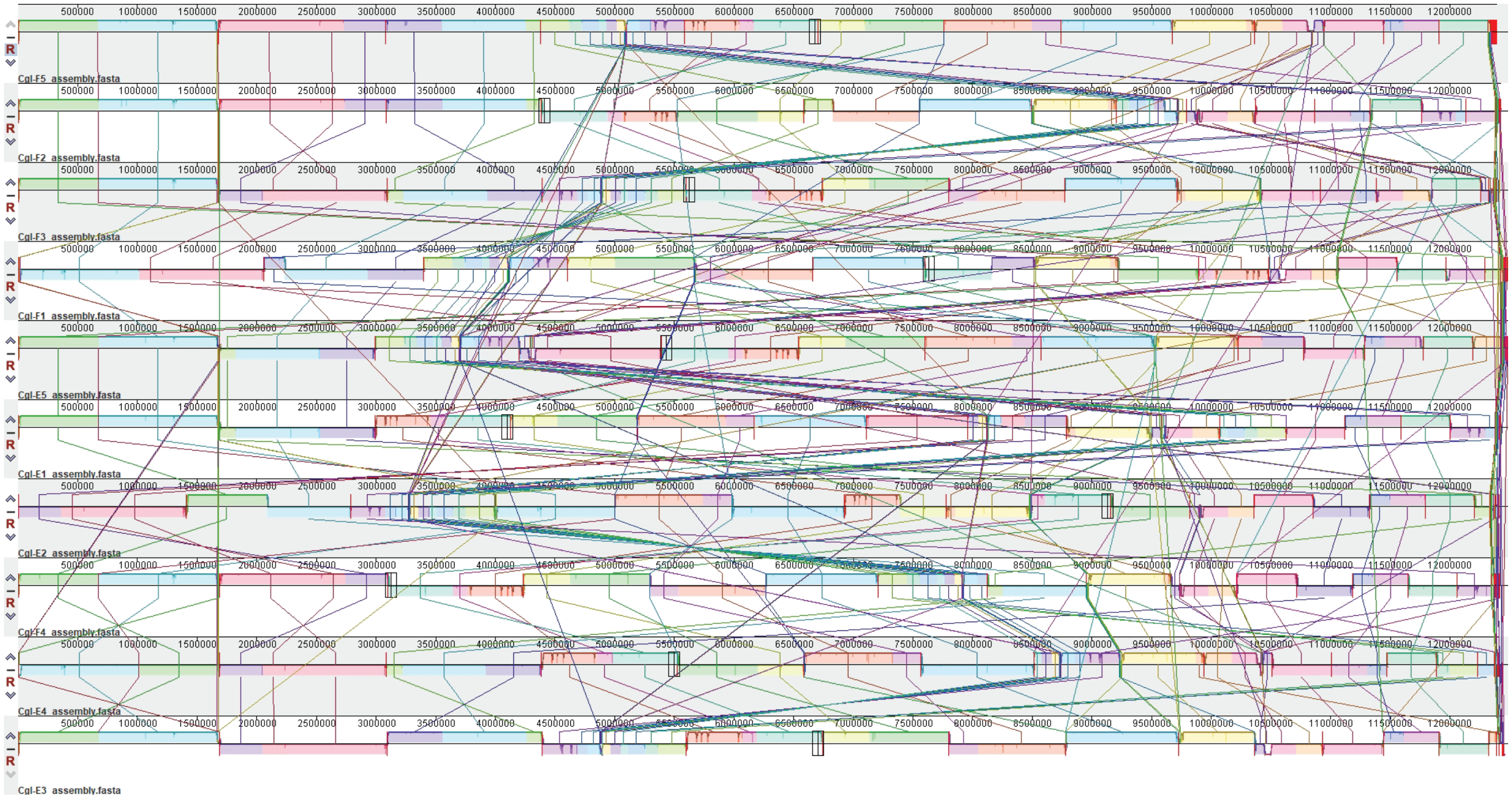
Cgl-F5 assembly.fasta
Cgl-F2 assembly.fasta
Cgl-F3 assembly.fasta
Cgl-F1 assembly.fasta
Cgl-E5 assembly.fasta
Cgl-E1 assembly.fasta
Cgl-E2 assembly.fasta
Cgl-F4 assembly.fasta
Cgl-E4 assembly.fasta
Cgl-E3 assembly.fasta

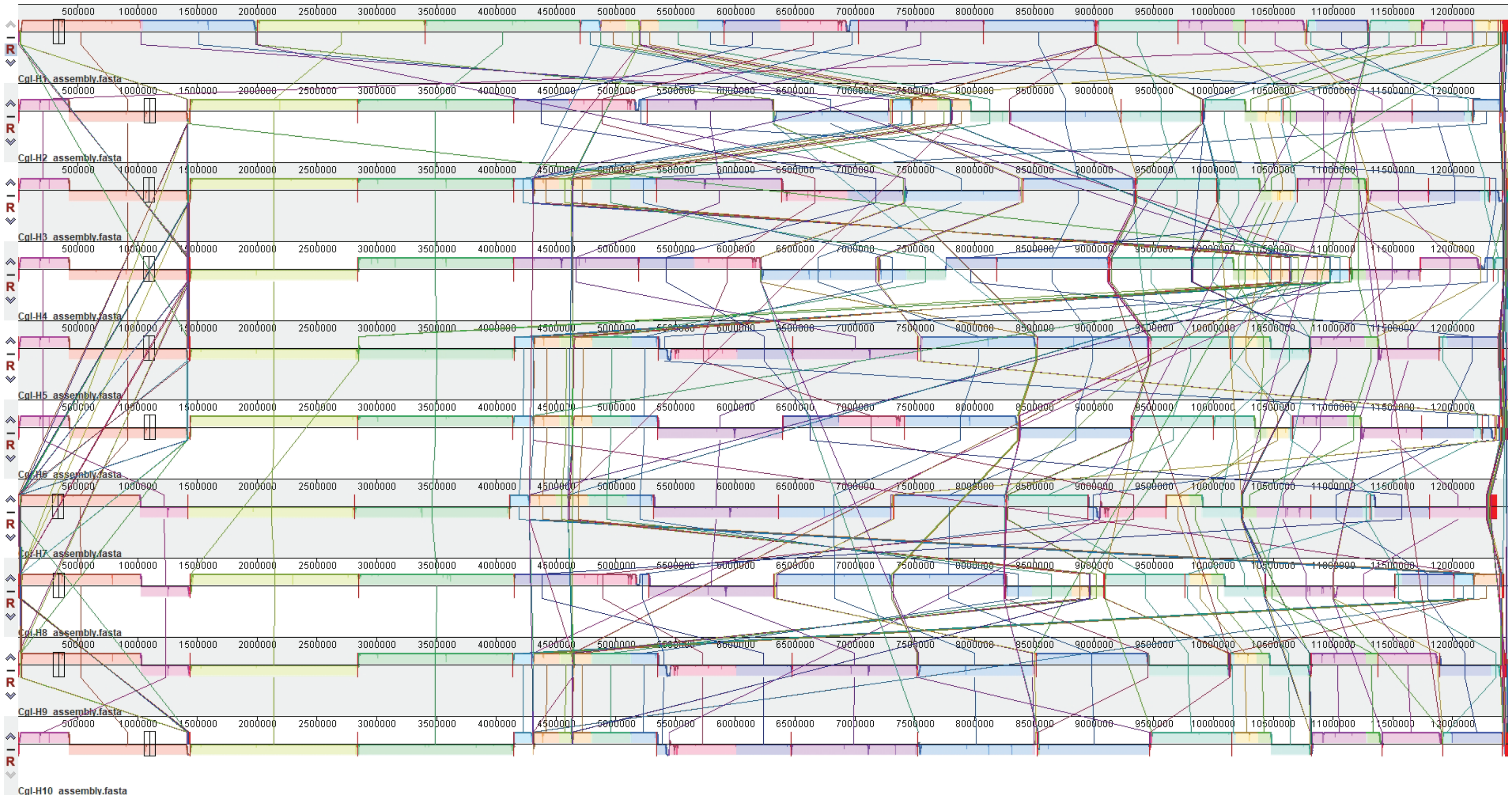
Cgl-H1 assembly.fasta
Cgl-H2 assembly.fasta
Cgl-H3 assembly.fasta
Cgl-H4 assembly.fasta
Cgl-H5 assembly.fasta
Cgl-H6 assembly.fasta
Cgl-H7 assembly.fasta
Cgl-H8 assembly.fasta
Cgl-H9 assembly.fasta
Cgl-H10 assembly.fasta

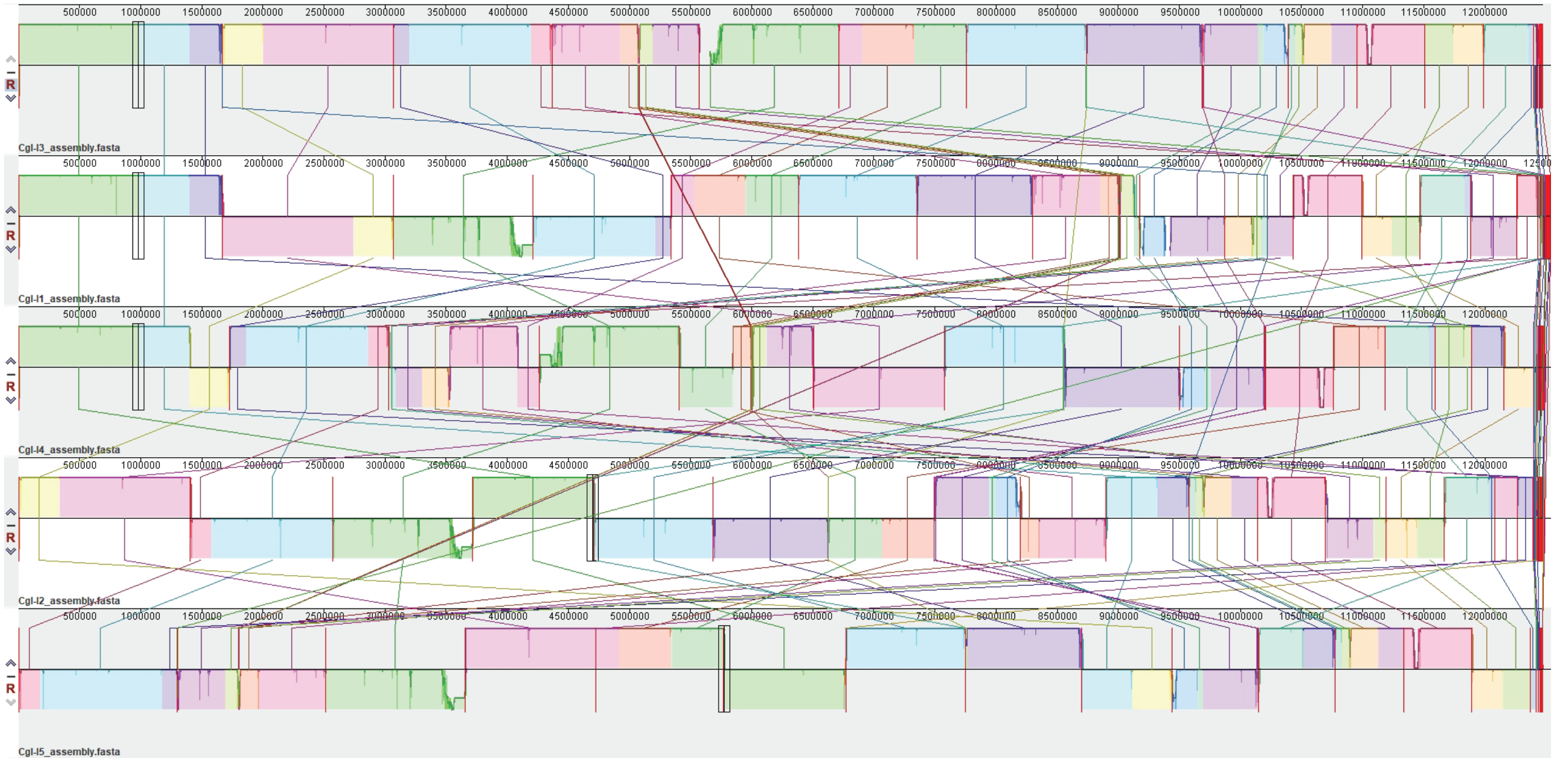
Cgl-I3_assembly.fasta
Cgl-I1_assembly.fasta
Cgl-I4_assembly.fasta
Cgl-I2_assembly.fasta
Cgl-I5_assembly.fasta
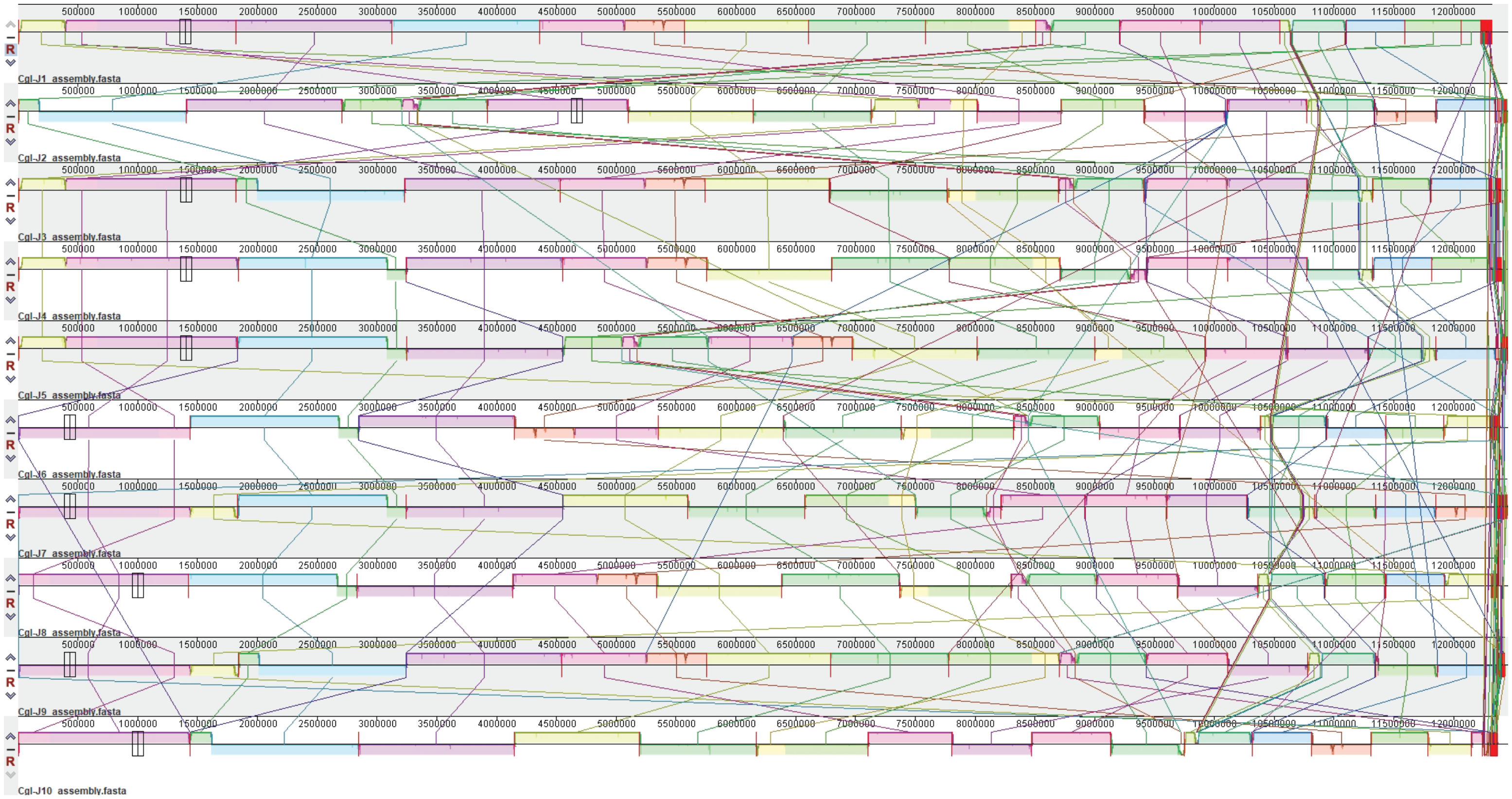
Cgl-J1_assembly.fasta
Cgl-J2_assembly.fasta
Cgl-J3_assembly.fasta
Cgl-J4_assembly.fasta
Cgl-J5_assembly.fasta
Cgl-J6_assembly.fasta
Cgl-J7_assembly.fasta
Cgl-J8_assembly.fasta
Cgl-J9_assembly.fasta
Cgl-J10_assembly.fasta

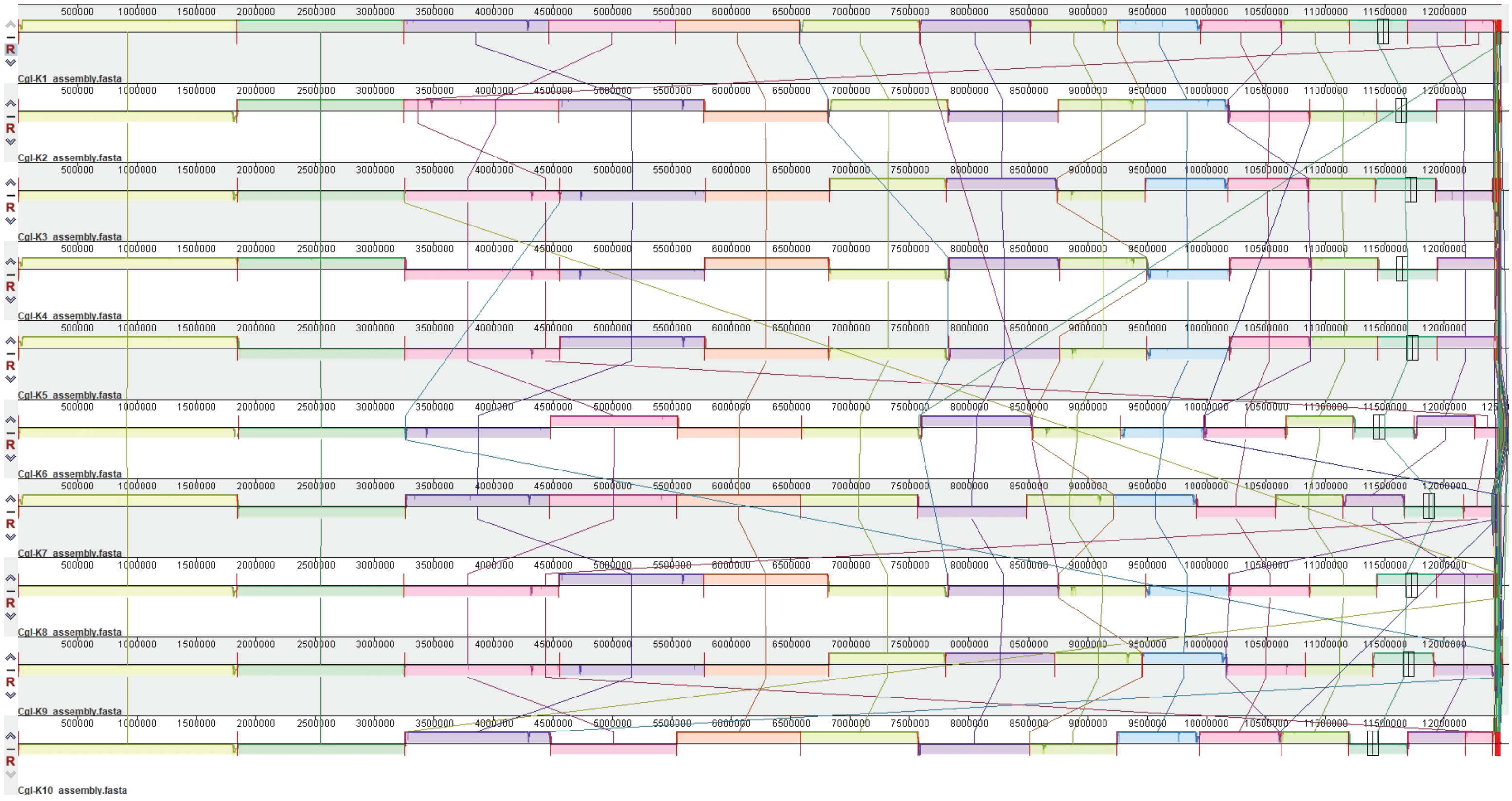

Cgl-K1_assembly.fasta
Cgl-K2_assembly.fasta
Cgl-K3_assembly.fasta
Cgl-K4_assembly.fasta
Cgl-K5_assembly.fasta
Cgl-K6_assembly.fasta
Cgl-K7_assembly.fasta
Cgl-K8_assembly.fasta
Cgl-K9_assembly.fasta
Cgl-K10_assembly.fasta

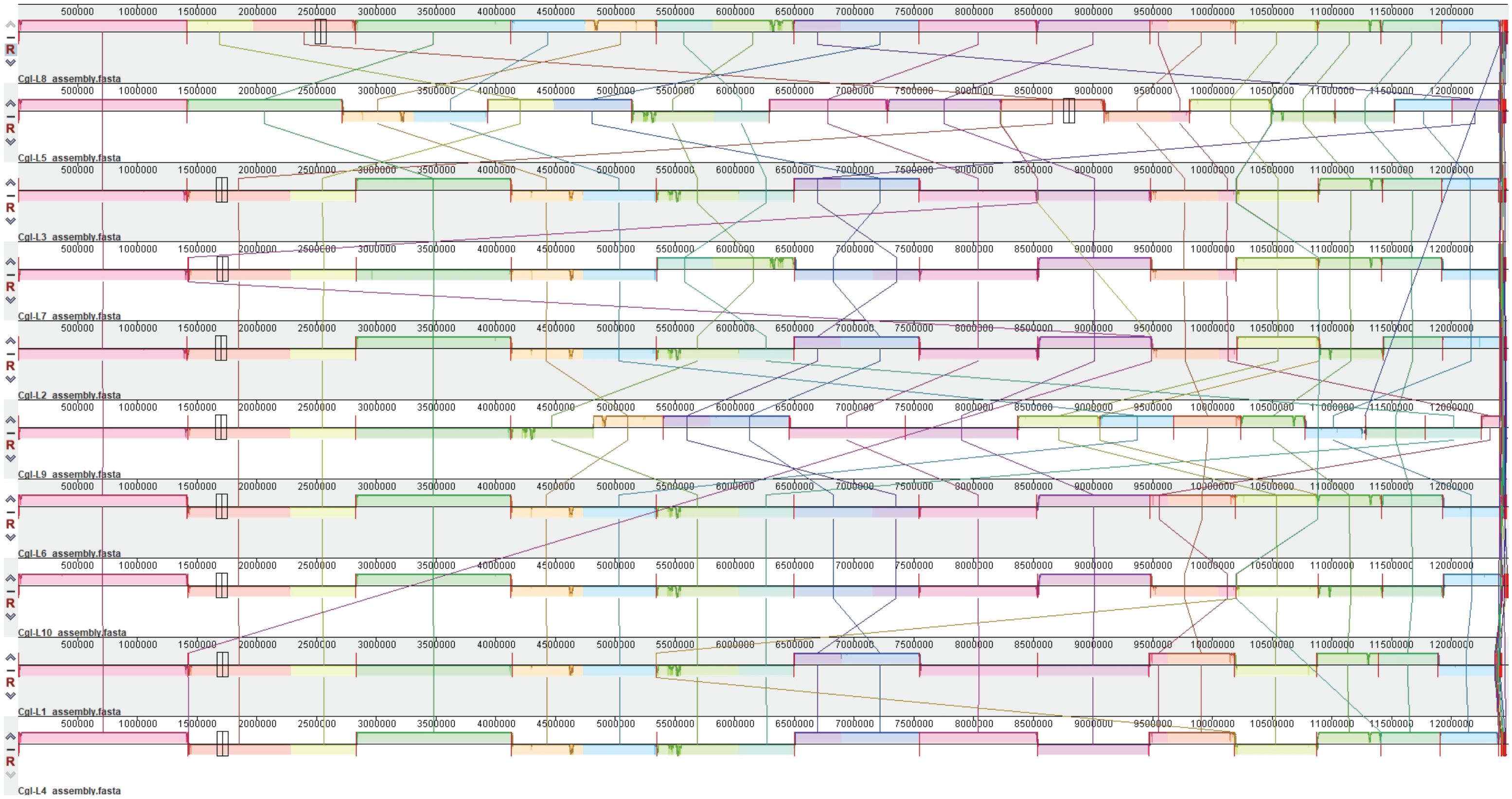

Cgl-L8_assembly.fasta
Cgl-L5_assembly.fasta
Cgl-L3_assembly.fasta
Cgl-L7_assembly.fasta
Cgl-L2_assembly.fasta
Cgl-L9_assembly.fasta
Cgl-L6_assembly.fasta
Cgl-L10_assembly.fasta
Cgl-L1_assembly.fasta
Cgl-L4_assembly.fasta

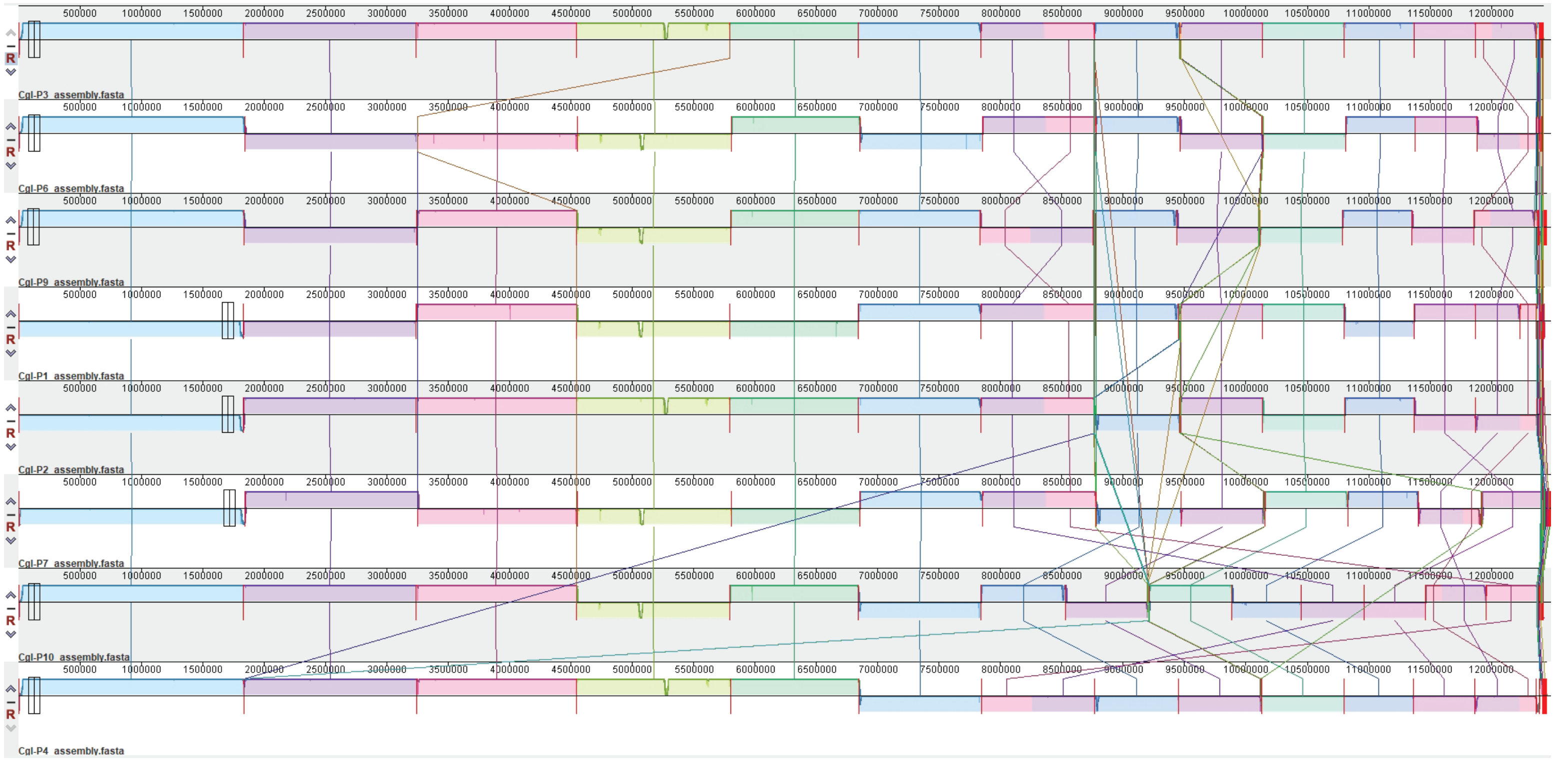
Cgl-P3_assembly.fasta
Cgl-P6_assembly.fasta
Cgl-P9_assembly.fasta
Cgl-P1_assembly.fasta
Cgl-P2_assembly.fasta
Cgl-P7_assembly.fasta
Cgl-P10_assembly.fasta
Cgl-P4_assembly.fasta

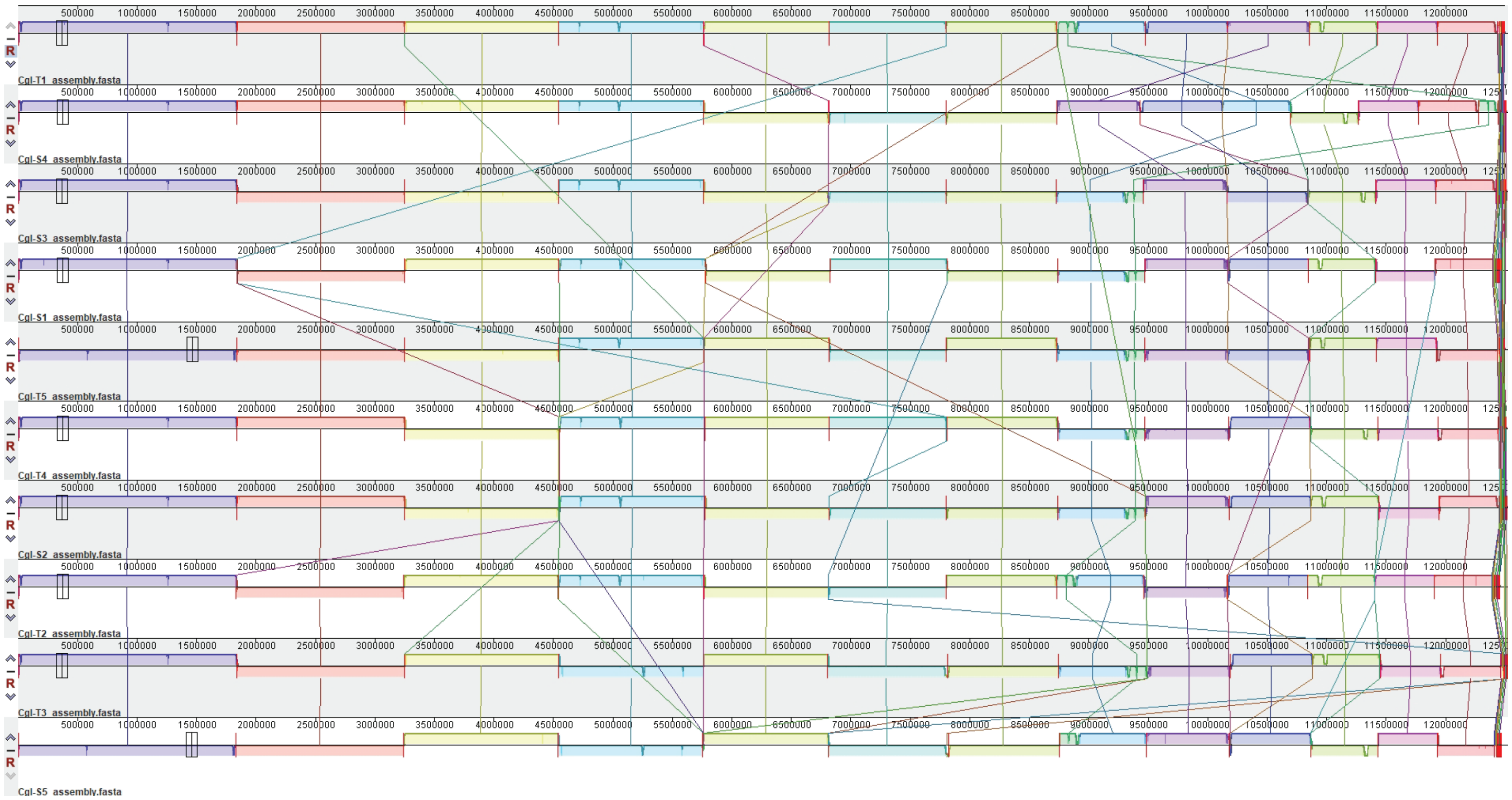
Cgl-T1_assembly.fasta
Cgl-S4_assembly.fasta
Cgl-S3_assembly.fasta
Cgl-S1_assembly.fasta
Cgl-T5_assembly.fasta
Cgl-T4_assembly.fasta
Cgl-S2_assembly.fasta
Cgl-T2_assembly.fasta
Cgl-T3_assembly.fasta
Cgl-S5_assembly.fasta

b Genomic similarity dot plots. Genomic similarity dot plots of pairwise comparisons showed chromosomal rearrangements in strains from 8 of 10 patients. No rearrangements were observed in strains from patients K or ST. For example, rearrangements in strain J1 include the following: Contig 1 of J1 is a fusion of contig 4 of J2 with a large segment of contig 7 of J2 (red lines); Contig 4 of J1 is a fusion of contigs 9 and 13 of J2 (red lines); and Contig 7 in J1 is a fusion of contig 9 and a small segment of contig 7 of J2 (red lines).

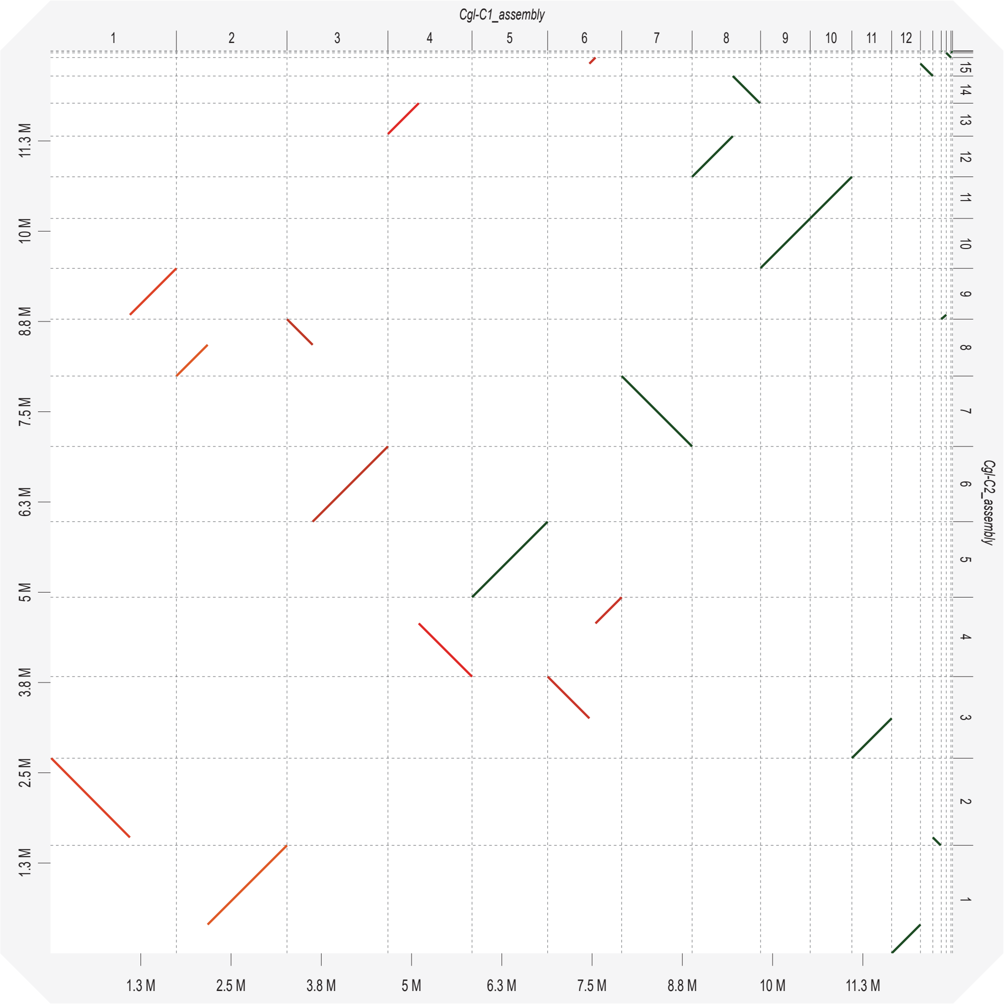

Cgl-C1_assembly
Cgl-C2_assembly
1.3 M
2.5 M
3.8 M
5 M
6.3 M
7.5 M
8.8 M
10 M
11.3 M

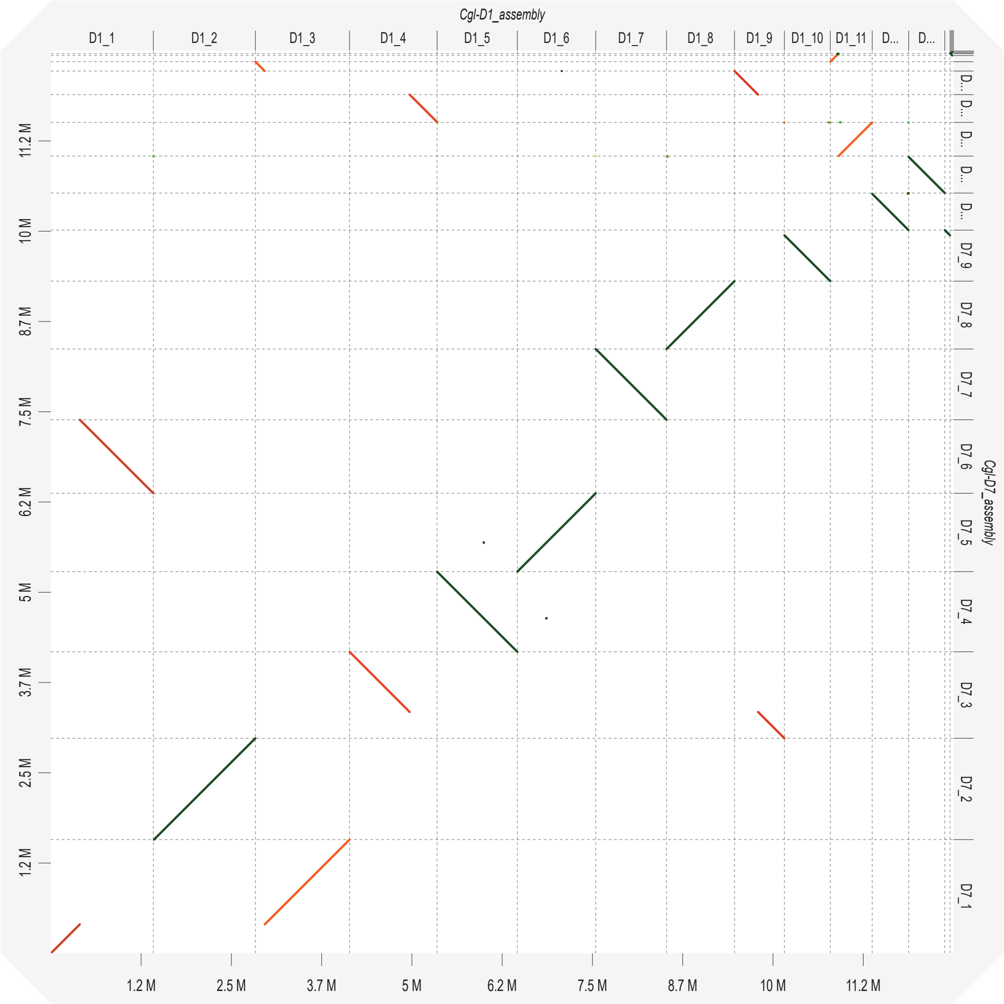

Cgl-D1_assembly
Cgl-D7_assembly
D1_1
D1_2
D1_3
D1_4
D1_5
D1_6
D1_7
D1_8
D1_9
D1_10
D1_11
D7_1
D7_2
D7_3
D7_4
D7_5
D7_6
D7_7
D7_8
D7_9
1.2 M
2.5 M
3.7 M
5 M
6.2 M
7.5 M
8.7 M
10 M
11.2 M

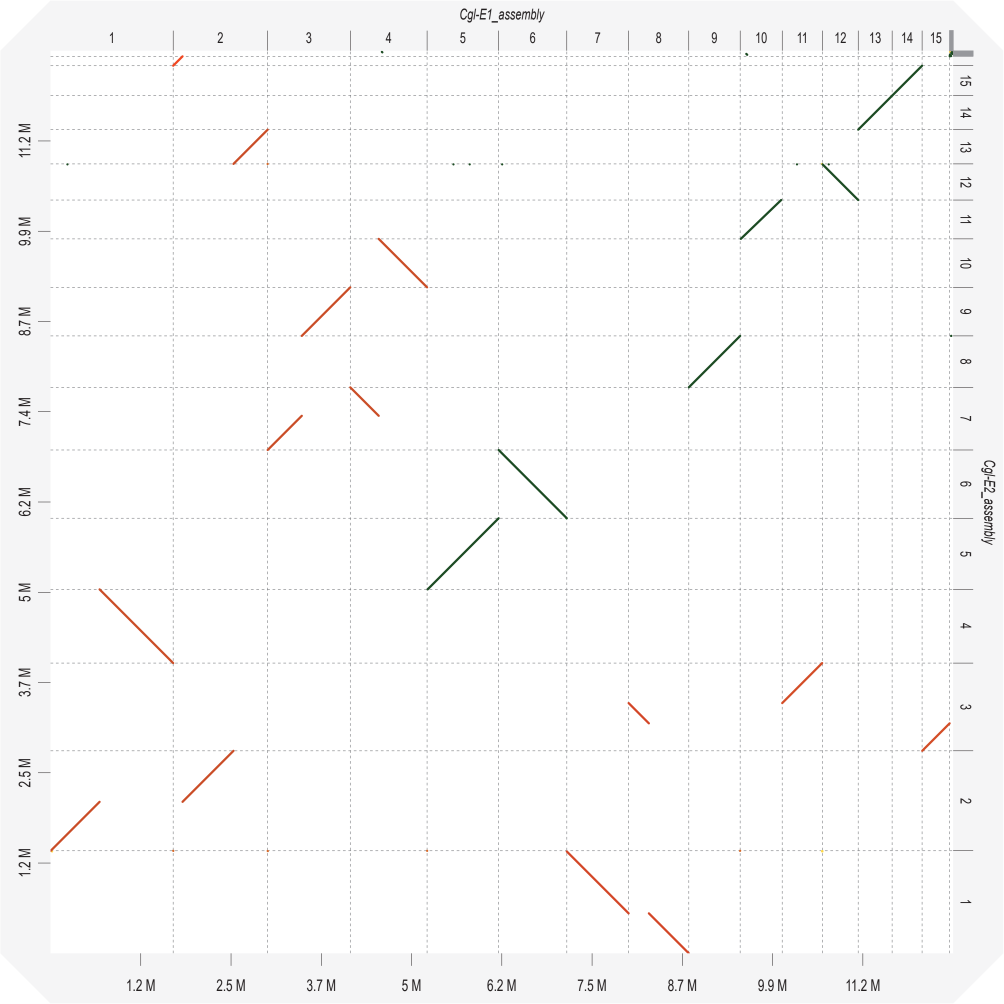
Cgl-E1_assembly
1 2 3 4 5 6 7 8 9 10 11 12 13 14 15
Cgl-E2_assembly
1 2 3 4 5 6 7 8 9 10 11 12 13 14 15
1.2 M
2.5 M
3.7 M
5 M
6.2 M
7.4 M
8.7 M
9.9 M
11.2 M
1.2 M
2.5 M
3.7 M
5 M
6.2 M
7.5 M
8.7 M
9.9 M
11.2 M

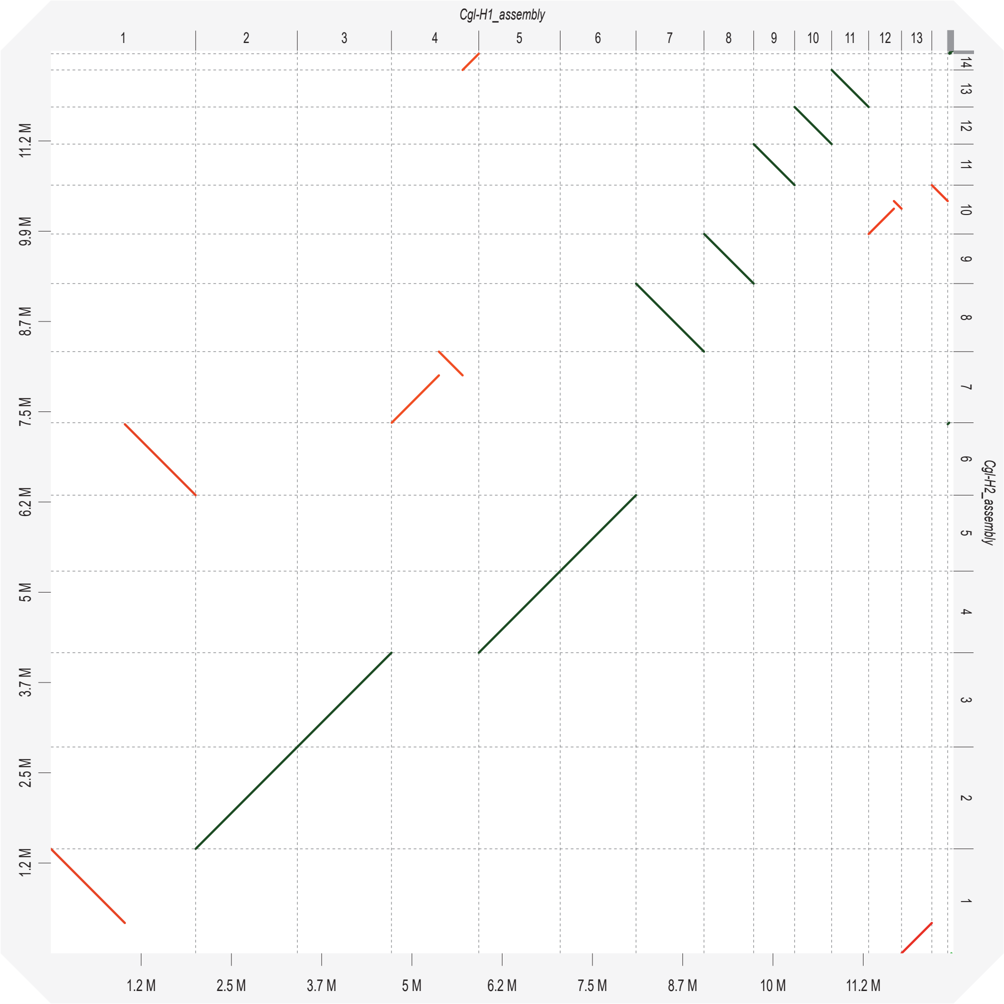
Cgl-H1_assembly
1 2 3 4 5 6 7 8 9 10 11 12 13
Cgl-H2_assembly
1 2 3 4 5 6 7 8 9 10 11 12 13 14
1.2 M
2.5 M
3.7 M
5 M
6.2 M
7.5 M
8.7 M
9.9 M
11.2 M
1.2 M
2.5 M
3.7 M
5 M
6.2 M
7.5 M
8.7 M
10 M
11.2 M

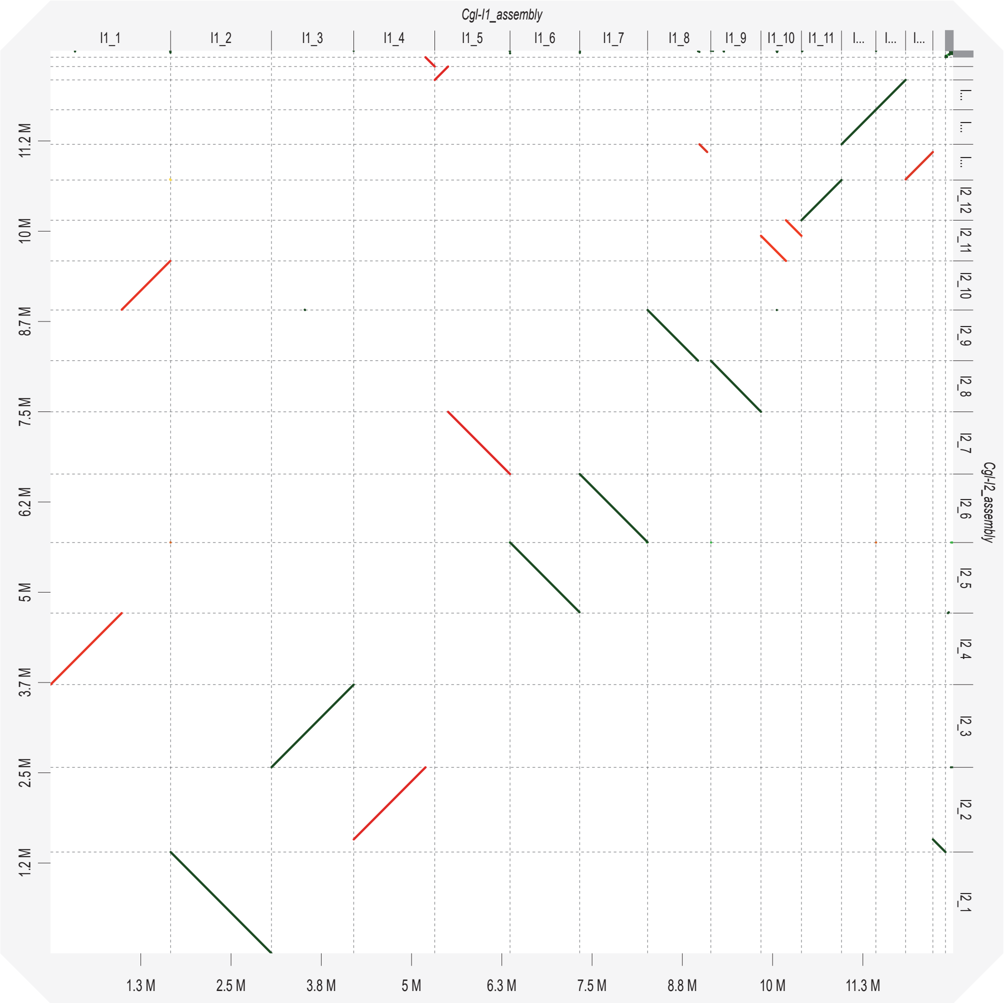
Cgl-I1_assembly
I1_1
I1_2
I1_3
I1_4
I1_5
I1_6
I1_7
I1_8
I1_9
I1_10
I1_11
Cgl-I2_assembly
I2_1
I2_2
I2_3
I2_4
I2_5
I2_6
I2_7
I2_8
I2_9
I2_10
I2_11
I2_12
1.3 M
2.5 M
3.8 M
5 M
6.3 M
7.5 M
8.8 M
10 M
11.3 M
1.2 M
2.5 M
3.7 M
5 M
6.2 M
7.5 M
8.7 M
10 M
11.2 M

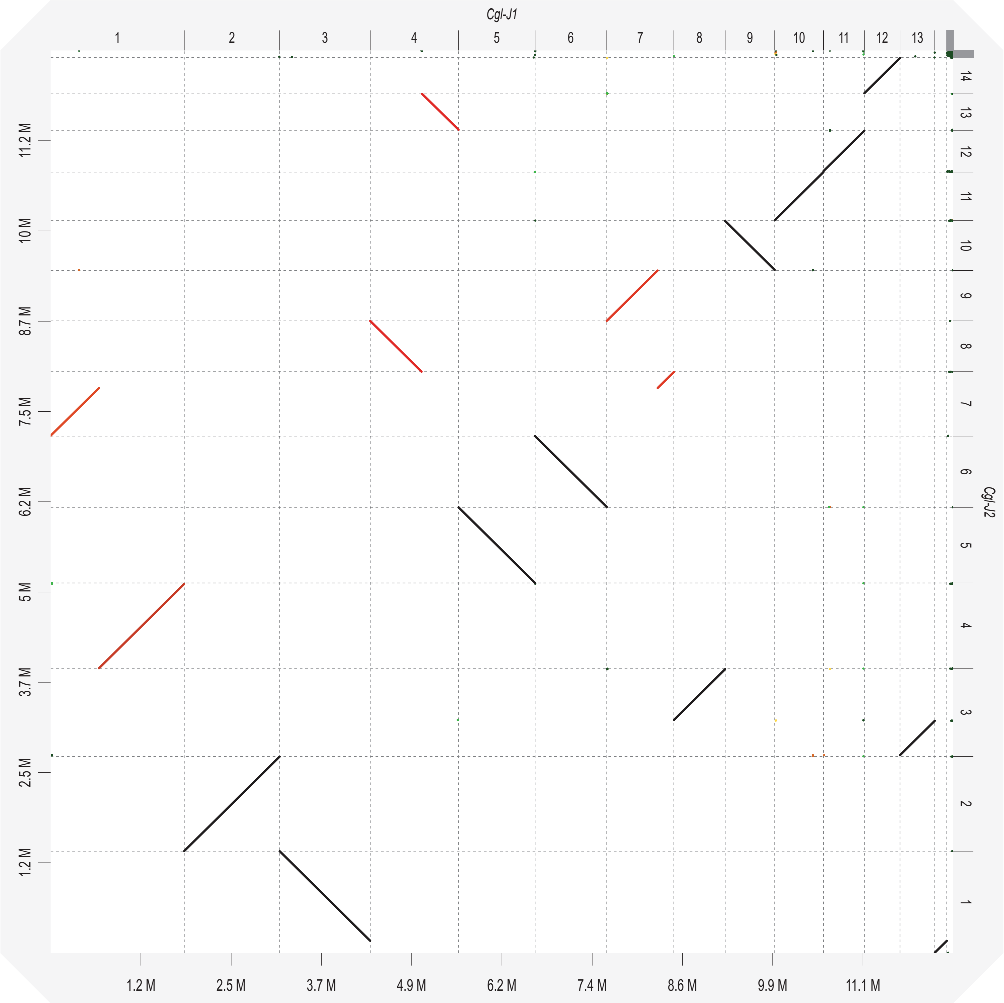
Cgl-J1
1
2
3
4
5
6
7
8
9
10
11
12
13
Cgl-J2
14
1.2 M
2.5 M
3.7 M
4.9 M
6.2 M
7.4 M
8.6 M
9.9 M
11.1 M
1.2 M
2.5 M
3.7 M
5 M
6.2 M
7.5 M
8.7 M
10 M
11.2 M

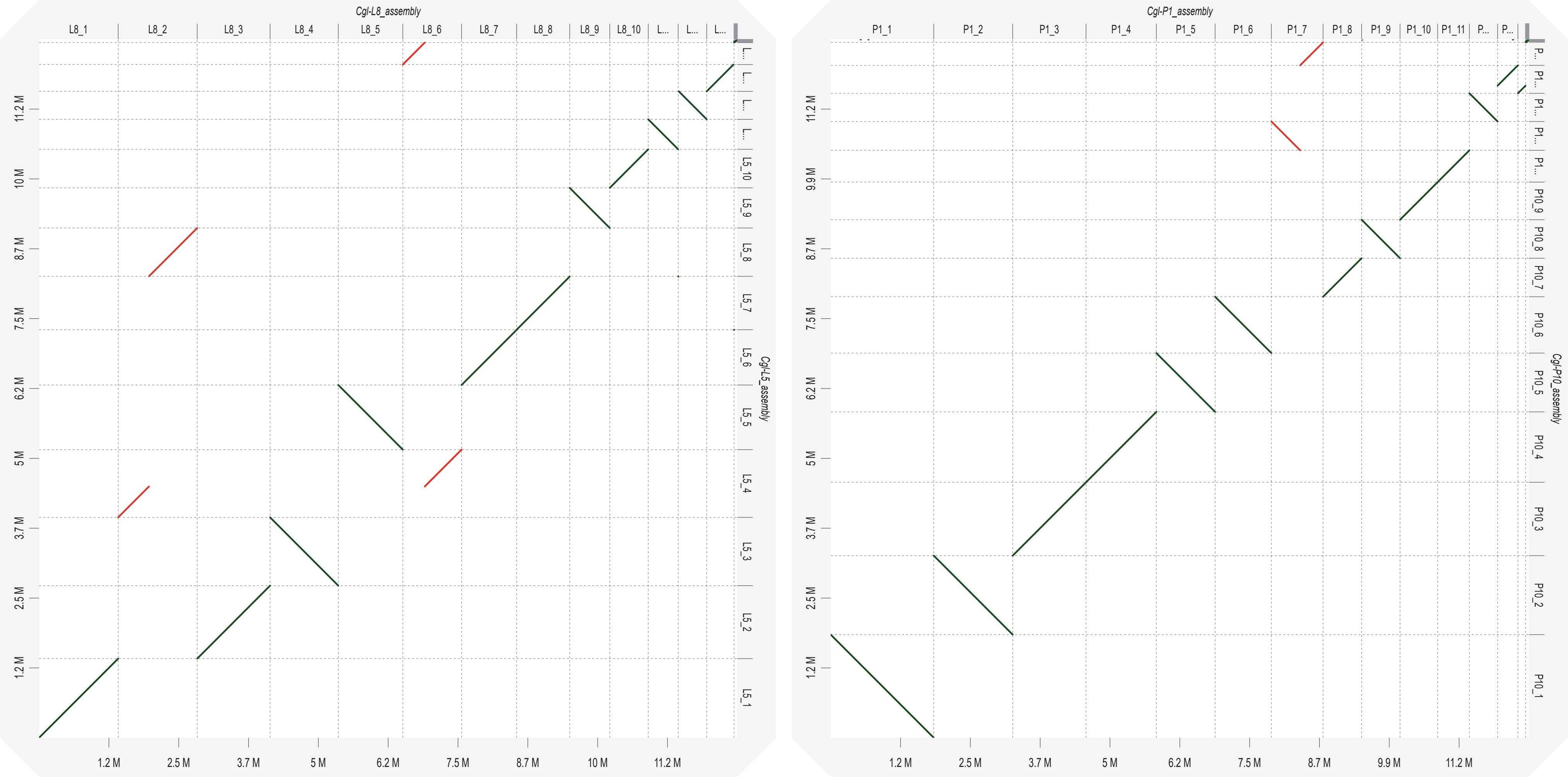
Cgl-L8_assembly
L8_1
L8_2
L8_3
L8_4
L8_5
L8_6
L8_7
L8_8
L8_9
L8_10
Cgl-L5_assembly
L5_10
L5_9
L5_8
L5_7
L5_6
L5_5
L5_4
L5_3
L5_2
L5_1
1.2 M
2.5 M
3.7 M
5 M
6.2 M
7.5 M
8.7 M
10 M
11.2 M
Cgl-P1_assembly
P1_1
P1_2
P1_3
P1_4
P1_5
P1_6
P1_7
P1_8
P1_9
P1_10
P1_11
Cgl-P10_assembly
P10_9
P10_8
P10_7
P10_6
P10_5
P10_4
P10_3
P10_2
P10_1
9.9 M
