# Supplementary material for: Genotypic diversity and unrecognized antifungal resistance among populations of Candida glabrata from positive blood cultures
Source: Nat Commun. 2023 Sep 22;14:5918. doi: 10.1038/s41467-023-41509-x (PMC10516878; doi:10.1038/s41467-023-41509-x)
Supplement: Supplementary file 3 — Description of Additional Supplementary Files [file 41467_2023_41509_MOESM3_ESM.pdf]

Description of Additional Supplementary Files

File Name: Supplementary Data 1
Description: Variants associated with non-synonymous mutations that discriminated within-patient strains (i.e., mutations found in at least one strain from a patient but not found in all strains, compared to C. glabrata CBS138).

File Name: Supplementary Data 2
Description: Datasets generated in this study
