# Supplementary material for: Genotypic diversity and unrecognized antifungal resistance among populations of Candida glabrata from positive blood cultures
Source: Nat Commun. 2023 Sep 22;14:5918. doi: 10.1038/s41467-023-41509-x (PMC10516878; doi:10.1038/s41467-023-41509-x)
Supplement: Supplementary file 6 — Reporting Summary [file 41467_2023_41509_MOESM6_ESM.pdf]

nature portfolio

Corresponding author(s): M. Hong NGUYEN
DBPR NCOMMS-23-11645

Last updated by author(s): Aug 18, 2023

Reporting Summary

Nature Portfolio wishes to improve the reproducibility of the work that we publish. This form provides structure for consistency and transparency in reporting. For further information on Nature Portfolio policies, see our Editorial Policies and the Editorial Policy Checklist.

Statistics

For all statistical analyses, confirm that the following items are present in the figure legend, table legend, main text, or Methods section.

n/a	Confirmed	
☐	☒	The exact sample size (*n*) for each experimental group/condition, given as a discrete number and unit of measurement
☒	☐	A statement on whether measurements were taken from distinct samples or whether the same sample was measured repeatedly
☐	☒	The statistical test(s) used AND whether they are one- or two-sided<br>*Only common tests should be described solely by name; describe more complex techniques in the Methods section.*
☒	☐	A description of all covariates tested
☒	☐	A description of any assumptions or corrections, such as tests of normality and adjustment for multiple comparisons
☐	☒	A full description of the statistical parameters including central tendency (e.g. means) or other basic estimates (e.g. regression coefficient) AND variation (e.g. standard deviation) or associated estimates of uncertainty (e.g. confidence intervals)
☐	☒	For null hypothesis testing, the test statistic (e.g. *F*, *t*, *r*) with confidence intervals, effect sizes, degrees of freedom and *P* value noted<br>*Give P values as exact values whenever suitable.*
☒	☐	For Bayesian analysis, information on the choice of priors and Markov chain Monte Carlo settings
☒	☐	For hierarchical and complex designs, identification of the appropriate level for tests and full reporting of outcomes
☒	☐	Estimates of effect sizes (e.g. Cohen's *d*, Pearson's *r*), indicating how they were calculated

Our web collection on statistics for biologists contains articles on many of the points above.

Software and code

Policy information about availability of computer code

Data collection	The following software was used for data collection: WGS (Illumina RTA v3.4.4); qPCR (BioRad CFX Maestro v2.3); Growth data (SPECTROstar Nano Mars v3.20 R2 28); flow cytometry (LSRFortessa with BD FACSDiva v8.0.2); TEM (AMT Capture Engine Software version 602.600.652
Data analysis	For data analysis, we used Nextflow V20.04.1 to built a pipeline integrating the following programs: bwa V0.7.17, GATK V4.2.2.0, snpeff V4.3t, and samtools V1.10. Further we used bcftools V1.15.1, fasttree V2.1.10, RaxML V0.9.0, Genome STRIP V2.0, flye V2.9, Pilon V1.23, and progressiveMauve V2.4.0, PubMLST (https://pubmlst.org/), Geneious D-Genies, iTol v6 58, Candida DB GO Term finder (http://www.candidagenome.org/help/goTermFinder.shtmlP), FlowJo v10.9, and SpectraMax I3x, Softmax Pro 6.4. For data presentation and statistical analysis, we used GraphPad Prism 9.1.2

For manuscripts utilizing custom algorithms or software that are central to the research but not yet described in published literature, software must be made available to editors and reviewers. We strongly encourage code deposition in a community repository (e.g. GitHub). See the Nature Portfolio guidelines for submitting code & software for further information.

Data

Policy information about availability of data

All manuscripts must include a data availability statement. This statement should provide the following information, where applicable:

- Accession codes, unique identifiers, or web links for publicly available datasets
- A description of any restrictions on data availability
- For clinical datasets or third party data, please ensure that the statement adheres to our policy

WGS data generated in this study have been deposited in the NCBI database under accession number SUB12947161 (https://submit.ncbi.nlm.nih.gov/subs/sra/SUB12947161/overview)

Human research participants

Policy information about studies involving human research participants and Sex and Gender in Research.

Reporting on sex and gender	Sex and gender were reported as percentages of the study population (N=10), and not linked to individual cases. Note that sex or gender was not considered in the study design. We included consecutive patients with positive blood cultures for Candida glabrata regardless of sex or gender
Population characteristics	Population characteristics are summarized in Table 1. Blood culture from 10 consecutive patients admitted to a single hospital with C. glabrata BSI were included. We cited potential limitations of this project in the Discussion, including the extent of clinical diversity we observed which may reflect particular features of our patient population, who were very ill and had extensive medical problems.
Recruitment	Patients with Candida glabrata bloodstream infection were identified as part of clinical care
Ethics oversight	This study complies with relevant ethical regulations. It was reviewed by the University of Pittsburgh Institutional Review Boards and approved through an "Expedited Review procedure" as published by the OHRP, 45 CFR 46.110 and FDA 21 CFR 56.110 (IRB protocol #22040147). The need for informed consent was waived for this project deemed to pose minimal risk to patients. We utilized remnant blood cultures from the UPMC Clinical Microbiology laboratory at the time they were discarded (the blood cultures were drawn purely for clinical care purpose). Clinical characteristics were identified through limited retrospective review of electronic medical records.

Note that full information on the approval of the study protocol must also be provided in the manuscript.

Field-specific reporting

Please select the one below that is the best fit for your research. If you are not sure, read the appropriate sections before making your selection.

[x] Life sciences [ ] Behavioural & social sciences [ ] Ecological, evolutionary & environmental sciences

For a reference copy of the document with all sections, see nature.com/documents/nr-reporting-summary-flat.pdf

Life sciences study design

All studies must disclose on these points even when the disclosure is negative.

Sample size	We selected 10 strains from blood culture bottles from 10 individual patients. No sample calculation was performed. Sample sizes were sufficient because we were able to identify genomic and phenotypic variation within individual blood culture bottles. For in vitro experiments, unless clearly stated, we used at least 3 independent repeats since this number is generally accepted with consistent results. For experiments with large margins of error, we used >3 experiments. For mice experiments, statistical analysis demonstrates that we will need at least 8 mice per group of mice infected with J1 or J9 to be able to detect at least 1-log difference in tissue burden between the 2 groups of mice, assuming the standard deviation of 0.5 with a power of 95% and an alpha of 0.02. In this study, we used a total of 16 mice (8 male and 8 female) mice.
Data exclusions	No data were excluded
Replication	Replication was performed for experiments using at least 3 biological replicates (unless clearly stated in the Results section). All attempts at replication were successful
Randomization	For clinical study, since this was a descriptive study, no randomization was performed (we included consecutive patients with Candida glabrata fungemia). For murine experiments, mice were randomly assigned to individual groups (infected with J1 or J9 strain) before the infection. The mice were sex and cage densities matched. For in vitro testing, the samples that we used were uniform, thus randomization was not relevant.
Blinding	Clinical data abstraction was performed by a research coordinator unfamiliar with the project design. For phenotypic experiments, results were collected by lab personnels who were unaware of which specific group of Candida strains were involved.

Reporting for specific materials, systems and methods

We require information from authors about some types of materials, experimental systems and methods used in many studies. Here, indicate whether each material, system or method listed is relevant to your study. If you are not sure if a list item applies to your research, read the appropriate section before selecting a response.

Materials & experimental systems

n/a	Involved in the study
x	Antibodies
	x Eukaryotic cell lines
x	Palaeontology and archaeology
	x Animals and other organisms
x	Clinical data
x	Dual use research of concern

Methods

n/a	Involved in the study
x	ChIP-seq
	x Flow cytometry
x	MRI-based neuroimaging

Eukaryotic cell lines

Policy information about cell lines and Sex and Gender in Research

Cell line source(s)	Hep-2 (ATCC CCL23) epithelial cell line was purchased from the American Type Culture Collection
Authentication	All cell lines were purchased from reputable sources, with whom we have longstanding relationships (e.g., American Type Culture Collection (ATCC)). Authentication was done using STR profiling. We scrupulously follow protocols for cell line initiation, maintenance, contamination prevention, and cryopreservation.
Mycoplasma contamination	Cell line was tested negative for Mycoplasma contamination
Commonly misidentified lines (See ICLAC register)	No commonly misidentified cell lines were used in the study

Animals and other research organisms

Policy information about studies involving animals; ARRIVE guidelines recommended for reporting animal research, and Sex and Gender in Research

Laboratory animals	CD-1 mice were purchased from Envigo, 4-6 weeks old and were acclimated for at least a week prior to experimentation. The mice were maintained in an SPF environment at an American Association for the Accreditation of Laboratory Animal Care-accredited animal facility at the University of Pittsburgh.
Wild animals	No wild animals were used in this study
Reporting on sex	The experiments were performed using equal numbers of male and female mice
Field-collected samples	Study did not involve samples collected from the field
Ethics oversight	Mouse experiments were performed under an animal study protocol approved by the University of Pittsburgh Animal Care and Use Committee (protocol # IS00018518). Mice were provided with water and a standard laboratory diet ad libitum. They were supplied with hardwood chips as bedding and housed in a temperature-controlled, air-conditioned room on a 12-hr light-dark cycle.

Note that full information on the approval of the study protocol must also be provided in the manuscript.

Flow Cytometry

Plots

Confirm that:

- [x] The axis labels state the marker and fluorochrome used (e.g. CD4-FITC).
- [x] The axis scales are clearly visible. Include numbers along axes only for bottom left plot of group (a 'group' is an analysis of identical markers).
- [ ] All plots are contour plots with outliers or pseudocolor plots.
- [ ] A numerical value for number of cells or percentage (with statistics) is provided.

Methodology

Sample preparation	Yeast cells in stationary phase (2E6/mL) were incubated with rhodamine 123 at a final concentration of 10 µg/mL for 30 min

at 37oC. To inhibit electron flow of the respiratory chain, cells were pre-incubated with 1 mM sodium azide for 2h before the addition of rhodamine 123. Cell fluorescence was quantified with a FACScan flow cytometer. For cell size measurement, unstained yeast cells were washed and evaluated using FACScan. Forward and side scatter gating (FSC-A and SSC-A, respectively) were recorded for 3,000 events.

Instrument	BD LSRFortessa
Software	FlowJo, Floreada.io
Cell population abundance	Yeast cells in stationary phase (2E6/mL)
Gating strategy	We identified population of interest by gating on size (forward/side-scatter), removed doublets, then by fluorophore. Gating analysis of flow cytometry data was performed using SpectroFlo® (version 3.0.0)., and the cell population of interest was identified with polygon gating.

☐ Tick this box to confirm that a figure exemplifying the gating strategy is provided in the Supplementary Information.
